# Mechanisms of Serotonergic Modulation of Glial Cell Functions in the Recovery Period After Spinal Cord Injury

**DOI:** 10.3390/cells15181667

**Published:** 2026-09-15

**Authors:** E. V. Nikiforova, A. Vetlugina, S. P. Konovalova, Y. I. Sysoev, P. E. Musienko

**Affiliations:** 1 Department of Neuroscience, Scientific Center for Genetics and Life Sciences, Sirius University of Science and Technology, 354340 Sochi, Russia; 2 Institute of Translational Biomedicine, St. Petersburg State University, 199034 St. Petersburg, Russia; 3 Pavlov Institute of Physiology, Russian Academy of Sciences (RAS), 199034 St. Petersburg, Russia; 4 Moscow Center for Advanced Studies, 20, Kulakova Str., 123592 Moscow, Russia

**Keywords:** serotonergic system, 5-HT, oligodendrocytes, remyelination, astrocytes, astrogliosis, microglia

## Abstract

**Highlights:**

**What are the main findings?**

- Serotonin exerts bidirectional effects on oligodendroglia: activation of the same 5-HT2A receptor can either promote differentiation (in immortalized lines) or induce apoptosis (in primary cultures), highlighting the critical role of the cellular model and microenvironment in determining the outcome.
- In glial cells other than oligodendrocytes, serotonin acts predominantly as an anti-inflammatory modulator: it suppresses the neurotoxic reactive astrocyte phenotype and NLRP3 inflammasome assembly via 5-HT2B and promotes the shift in microglia from a pro-inflammatory toward a reparative/immunoregulatory phenotype through 5-HT1A, 5-HT2A, 5-HT2B, and 5-HT7 receptors.

**What are the implications of the main findings?**

- The pleiotropic and cell-type-specific nature of serotonergic signaling necessitates the development of highly selective receptor ligands to avoid opposing effects on different glial populations.
- The efficacy of serotonergic therapy in SCI is critically time-dependent: interventions that are beneficial in the chronic phase (promoting remyelination and regeneration) may be counterproductive in the acute phase due to excitotoxic and pro-apoptotic effects of excessive serotonin.

**Abstract:**

Spinal cord injury (SCI) leads to severe disability in patients, and one of the key obstacles to the recovery of lost functions is inefficient axonal remyelination. The high sensitivity of oligodendrocytes to secondary damage, the formation of a glial scar, and chronic neuroinflammation sustained by pro-inflammatory/neurotoxic microglia create a physical and chemical barrier to neuroplasticity processes. This review summarizes current knowledge on the ability of the serotonergic system to modulate the functional state of the main types of glial cells: oligodendrocytes, astrocytes, and microglia. It is shown that oligodendrocyte precursor cells (OPCs) and mature oligodendrocytes express 5-HT_1A_, 5-HT_2A_, and 5-HT_7_ receptors, whose activation can either stimulate differentiation or suppress migration or induce apoptosis, depending on the developmental stage and microenvironment. In astrocytes, serotonin, predominantly via 5-HT_2B_ receptors, limits pro-inflammatory transformation, reduces NLRP3 inflammasome activity, and stimulates BDNF production, glycogenolysis, and lactate release. In microglia, serotonergic stimulation (via 5-HT_1A_, 5-HT_2A_, 5-HT_2B_, and 5-HT_7_ receptors) attenuates the pro-inflammatory phenotype by suppressing production of TNF-α and IL-1β. To date, the proposed therapeutic strategies include: the use of 5-HT receptor agonists/antagonists and transplantation of serotonergic cells (hNT2.19 and RN46A-B14 lines), which reduce neuropathic pain and promote motor function recovery in rodents after SCI. The key limitations for the effective translation of these findings into clinical practice are the heterogeneity of effects of different serotonin receptor subtypes, the dependence of outcomes on the time after injury, and pronounced species specificity. Thus, the serotonergic system represents a promising but complex target for inducing neuroplasticity processes; success requires the development of subtype-selective ligands and treatment protocols that take into account the stages of pathological processes in SCI.

## 1. Introduction

Spinal cord injury (SCI) is a major medical and social burden and a leading cause of disability, affecting an estimated 250,000–500,000 people worldwide annually [1]. To date, no methods exist that can fully restore functions lost after SCI. Under these circumstances, remyelination takes on particular importance: although it is not an emergency treatment measure, it can provide partial recovery of sensorimotor and autonomic functions [2,3].

After injury, oligodendrocytes, the principal myelinating cells of the central nervous system (CNS), die en masse because of their high sensitivity to stress-induced factors. Although their precursors (OPCs) retain the capacity to proliferate and differentiate [4], the inflammatory microenvironment suppresses their maturation, making remyelination in the chronic phase slow and inefficient. Stimulating remyelination is regarded as a promising strategy: experimental models have shown that OPC transplantation and pharmacological stimulation of endogenous remyelination accelerate functional recovery after SCI [2,3]. In addition, astrocytes and microglia play a key role in forming the glial scar and sustaining inflammation. For this reason, shifting them pharmacologically or genetically toward an anti-inflammatory phenotype is viewed as a promising adjunctive strategy that can attenuate secondary damage and create conditions for durable neurorehabilitation.

Among the promising targets of neuromodulatory therapy, the serotonergic system stands out [5]; it modulates the activity of the spinal locomotor generator. Selective stimulation of serotonergic receptors promotes recovery of motor function after SCI [6]. However, almost all studies have focused exclusively on the functions of neuronal populations, neglecting effects on glial cells (OPCs, oligodendrocytes, astrocytes, and microglia). The key gap, therefore, is the lack of data on how the serotonergic system regulates the functional state of glial cells under SCI conditions, in particular OPC differentiation, the phagocytic activity of microglia, and reactive astrogliosis. Filling this gap is necessary for developing comprehensive post-SCI treatment strategies that combine neuronal and glial modulation.

The aim of the present review is to summarize current data on the role of the serotonergic system in modulating glial cells (OPCs, oligodendrocytes, astrocytes, and microglia), with an emphasis on the potential to induce remyelination, and to analyze the limitations and risks associated with the pleiotropic effects of serotonin.

## 2. Glial Responses After SCI

### 2.1. Post-Traumatic Response of Oligodendroglia

In SCI, the primary mechanical damage to tissue is aggravated by the triggering of a secondary inflammatory cascade [7]. The cells most sensitive to secondary damaging factors (excitotoxicity, oxidative stress, extracellular ATP, pro-inflammatory cytokines, and chemokines) are oligodendrocytes, the principal myelinating cells of the CNS [8,9]. Their mass death leads to progressive demyelination and degeneration of nerve fibers [10]. Unlike the Schwann cells of the peripheral nervous system, each of which myelinates only a single axon, one oligodendrocyte simultaneously forms myelin segments on 60–200 axons [11]; as a result, the death of even a single oligodendrocyte leads to the demyelination of many axons [12].

Traditionally, the function of oligodendrocytes was considered to be exclusively confined to the formation of the myelin sheath, which provides rapid propagation of action potentials along the nerve fiber by reducing membrane electrical capacitance and decreasing the energy expenditure required for restoring potassium and sodium gradients [13,14,15]. However, current evidence indicates that the functional repertoire of oligodendrocytes is considerably broader and encompasses neurotrophic support [16,17], immunomodulation [18,19,20], and participation in neural network plasticity through mechanisms of adaptive myelination [21].

At the epicenter of SCI, primary injury causes immediate rupture of oligodendrocyte membranes and their myelin sheaths, resulting in the loss of ionic homeostasis and the activation of necrotic cell death pathways [10,22,23]. Damage-associated molecular patterns (DAMPs) released from the cytoplasm trigger a secondary inflammatory cascade, recruiting macrophages and exacerbating tissue destruction. For instance, ATP, through binding to purinergic receptors (P2X, P2Y), activates calcium-dependent signaling pathways that lead to oligodendrocyte apoptosis [24,25,26].

**Figure 1:**
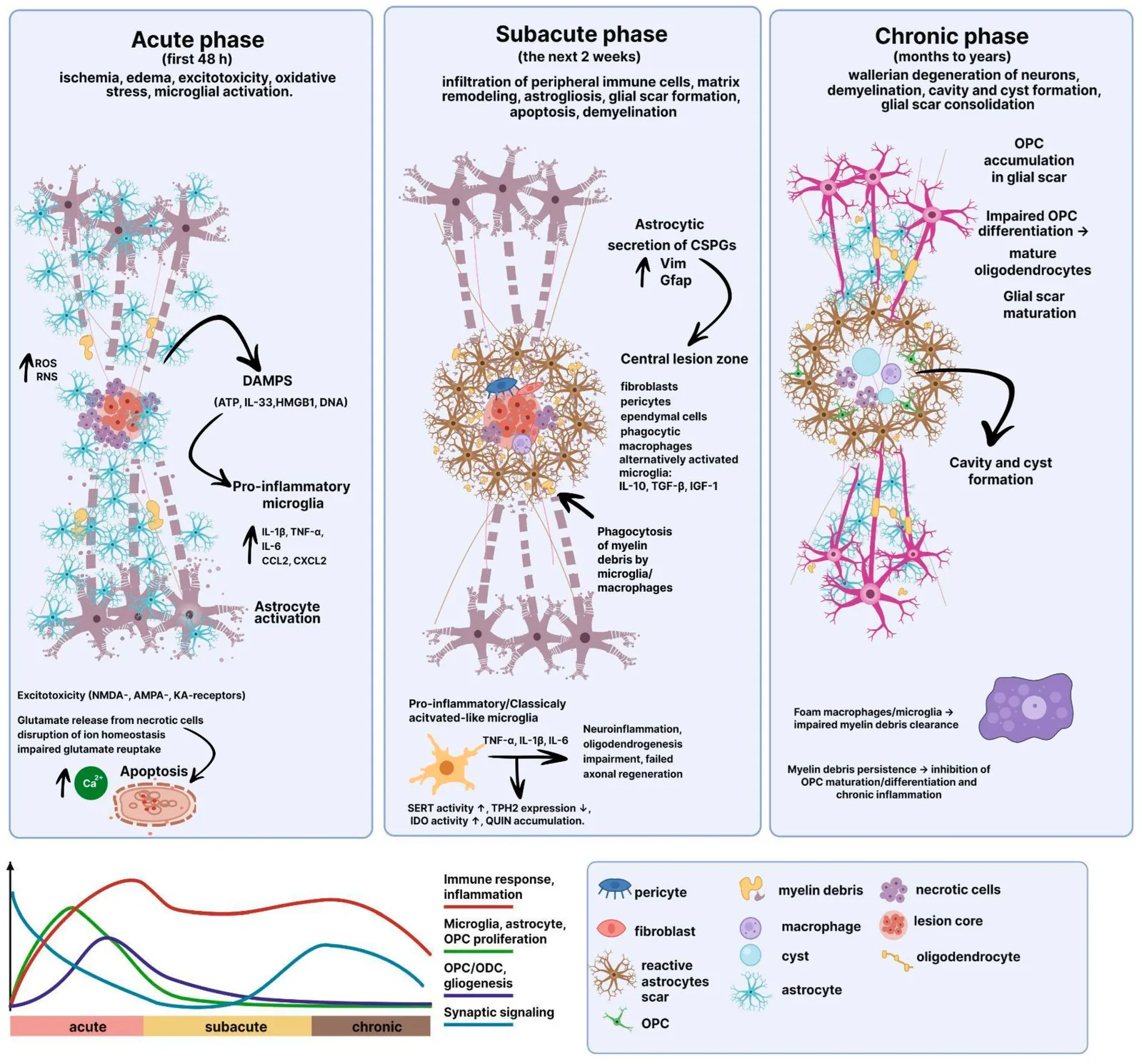
Stages of secondary damage to nervous tissue after SCI. Temporal dynamics of the cellular response after SCI. ROS–Reactive Oxygen Species, RNS–Reactive Nitrogen Species, DAMPS–Damage-Associated Molecular Patterns, ATP–Adenosine Triphosphate, IL-33–Interleukin-33, HMGB1–High Mobility Group Box 1, DNA–Deoxyribonucleic Acid, IL1β–Interleukin-1 beta, TNF-α–Tumor Necrosis Factor alpha, IL-6–Interleukin-6, CCL2–C-C Motif Chemokine Ligand 2, CXCL2–C-X-C Motif Chemokine Ligand 2, NMDA–N-methyl-D-aspartate, AMPA–α-amino-3-hydroxy-5-methyl-4-isoxazolepropionic acid, KA–Kainate receptors, Vim–Vimentin, Gfap–Glial Fibrillary Acidic Protein, SERT–Serotonin Transporter, TPH2–Tryptophan Hydroxylase 2, IDO–Indoleamine 2,3-Dioxygenase, QUIN–Quinolinic Acid.

Following the primary insult, a cascade of secondary processes predictably unfolds, which is conventionally divided into three phases: acute, subacute, and chronic. The acute phase, lasting the first 48 h post-injury, is characterized by ischemia, edema, excitotoxicity, oxidative stress, and microglial activation. The subacute phase (the subsequent two weeks) is characterized by infiltration of the lesion site by cells of the peripheral immune system (macrophages and leukocytes), matrix remodeling, the development of astrogliosis with glial scar formation, and ongoing cell apoptosis and demyelination. The chronic phase, which may persist for months or even years, involves Wallerian degeneration of axons, chronic demyelination, cavity and cyst formation, and the final consolidation of the glial scar, which segregates the lesion core from the surrounding healthy tissue [27] (Figure 1).

During the acute phase, energy deficit and ionic imbalance induce excitotoxic oligodendrocyte injury. Prolonged stimulation of AMPA and kainate receptors has been shown to predominantly mediate both caspase-dependent and caspase-independent pathways of oligodendrocyte death [28,29]. Beyond direct Ca^2+^-dependent damage, AMPA receptor activation in oligodendrocytes triggers Ca^2+^-mediated cytosolic acidification and subsequent release of Zn^2+^ from intracellular stores (mitochondria and metalloproteins), which exacerbates mitochondrial dysfunction, reactive oxygen species (ROS) production, and cell death [30].

It is also important to emphasize that ionotropic glutamate receptors are unevenly distributed across the oligodendrocyte surface; thus, excessive glutamate release elicits differential effects: NMDA receptors, localized to the myelin sheath, induce process swelling, compact myelin deformation, and subsequent nerve fiber demyelination [31], whereas AMPA and KA receptors, predominantly located on the soma, trigger apoptosis via caspase-dependent and -independent routes.

In various rodent models of SCI, oligodendrocytes undergo massive cell death during the first minutes after injury and throughout the subsequent acute and subacute phases, with apoptotic bodies still detectable for nearly three weeks [22,32,33,34]. This pronounced vulnerability of oligodendrocytes is rooted in their metabolic peculiarities [35]: high metabolic activity, high iron content serving as a cofactor for myelination enzymes, and low antioxidant capacity [36,37]. This deficiency in antioxidant defenses renders oligodendrocytes unable to neutralize free radicals, ultimately leading to irreversible mitochondrial damage, disruption of myelin membranes, and the initiation of apoptosis.

In the subacute period, the death of mature oligodendrocytes continues; however, the mechanisms of cell death shift towards apoptosis, in contrast to the necrotic death characteristic of the acute phase [4]. Concurrently, repair mechanisms are activated, involving the proliferation and migration of oligodendrocyte precursor cells (OPCs) [38,39]. OPC proliferation remains highest during the first two weeks post-injury [40,41,42]. OPCs are recruited to the injury zone from adjacent white matter regions and begin the process of differentiation into mature oligodendrocytes. Notably, as the glial scar matures, OPCs accumulate along the lesion boundary, contributing to its reinforcement; however, OPC differentiation into mature oligodendrocytes is arrested within the glial scar [43,44]. By day 3 post-injury, oligodendrocyte numbers fall below baseline levels, but due to active oligodendrogenesis, they increase approximately threefold by day 14 [45,46], compensating for the apoptotic death of mature oligodendrocytes, which peaks around the second week after trauma [22].

A critical determinant of OPC fate is the microenvironment of the injured tissue. Pro-inflammatory cytokines such as TNF-α and IL-1β, persisting extracellular myelin containing inhibitors (MAG, Nogo, OMgp) [47,48], and the pro-inflammatory/neurotoxic phenotype of macrophages all suppress precursor differentiation and limit remyelination. Furthermore, T cells indirectly induce oligodendrocyte apoptosis through the release of molecules that activate death receptors (DR) under the influence of TNF-α, IL-2, and IFN-γ, or via direct attack leading to cell lysis [49]. Conversely, anti-inflammatory molecules derived from macrophages and microglia promote OPC maturation and myelin repair.

The chronic phase of spinal cord injury, occurring months after the insult, is characterized by the establishment of persistent structural and molecular barriers to regeneration [50]. During this period, a reduction in the total number of mature oligodendrocytes is observed, alongside the persistence of an OPC population, a significant portion of which remains undifferentiated [50]. Although remyelination is possible in the chronic phase, it is typically incomplete and functionally insufficient [51,52], due to prolonged inflammation, metabolic disturbances, and the presence of inhibitory factors within the extracellular matrix, including molecules such as tenascin-C and chondroitin sulfate proteoglycans [51,53]. The glial and fibrotic scars create not only a physical obstacle to regeneration but also establish a biochemical milieu unfavorable for OPC differentiation [53]. In the chronic phase, signals promoting the astrocytic conversion of OPCs are enhanced. Among these signals, bone morphogenetic proteins (BMPs) have been identified [54,55,56,57], with increased BMP expression correlating with a reduced differentiation potential of OPCs [42].

### 2.2. Reactive Astrogliosis: Formation of a Physical and Chemical Barrier

After SCI, astrocytes undergo a pronounced transformation termed “reactive astrogliosis,” as a result of which they activate the expression of genes for the extracellular matrix (*Icam1*, *Lgals1*, *Vcan*), the immune response (*Cxcl1*, *Cxcl2*, *Cxcl10*, *Il6*, *Lif*), the cytoskeleton and intermediate filaments (*Gfap*, *Vim*), metabolism (*Gfpt2*, *Hk2*, *Cyb5r3*), and others [58,59], and they form a glial scar [59,60,61].

The glial scar, which develops during the subacute phase of injury and attains structural maturity in the chronic period, is characterized by a dual functional role. On one hand, it forms a physical barrier that restricts the dissemination of the inflammatory infiltrate and secondary damaging factors into the adjacent intact tissue [62]. On the other hand, glial scar cells, by secreting a broad spectrum of signaling molecules and inhibitory mediators, establish a chemical barrier that impedes axonal growth and successful regeneration [63,64]. Glial scar formation is a multi-step process initiated by signaling molecules IL-1α, TNF-α, and C1q, which are released by activated microglia [65], as well as by signals from M2-phenotype macrophages. These factors trigger astrocyte activation and enhance their chemotaxis toward the lesion site [66]. Within 1–2 days, astrocytes begin to proliferate actively; their proliferative peak occurs around the first week, after which the rate of division declines, and by day 14, their proliferation virtually ceases.

Reactive astrocytes secrete a wide array of intercellular signaling molecules, including cytokines [58], chemokines [67,68], metabolic energy substrates [69], toxic amino acids [70], and growth factors [71,72,73]. These molecules exert effects both on the astrocytes themselves and on surrounding cells (neurons, OPCs, and microglia).

During reactive astrogliosis, enhanced and synchronized calcium waves are observed [74,75], which not only reflect the hyperactivity of the astrocytic syncytium but also stimulate excessive secretion of gliotransmitters, primarily glutamate and ATP. These molecules serve as key mediators of excitotoxicity, inducing neuronal membrane damage, disrupting synaptic transmission, and ultimately leading to the death of neighboring cells.

During the first week post-injury, reactive astrocytes migrate toward the lesion epicenter, extending their processes to form a dense meshwork around the injured area and interweaving with processes of reactive microglia and NG_2_-glia. This results in the formation of a barrier that mechanically isolates the damaged region and prevents the spread of pro-inflammatory mediators into undamaged areas [76,77,78]. The central lesion core is populated by fibroblasts (originating from perivascular cells), pericytes, ependymal cells, and phagocytic macrophages [79], which together form a fibrotic scar.

It is in the containment of the damaged area during the early post-injury phase that the protective role of the astroglial scar resides. Experimental models involving suppression of astrocyte activation or complete ablation of the glial scar have demonstrated enhanced propagation of damage into intact tissues and worsened sensorimotor functional recovery. For instance, in mice with knockout of the *Gfap* and *Vim* genes, less dense scars are formed, often accompanied by hemorrhages and expansion of the lesion area [58,80]. Conversely, the glial scar inhibits axonal sprouting and regeneration. Among the key components of the glial barrier are chondroitin sulfate proteoglycans (CSPGs), which are actively secreted by reactive astrocytes and other cells of the astroglial scar [59,81]. CSPGs include aggrecan, versican, neurocan, brevican, and phosphacan—extracellular matrix components that suppress axonal growth, neural circuit restoration, and OPC maturation [82,83,84,85], collectively leading to neuronal degeneration and a worsened recovery prognosis.

Glial scar formation represents a complex process involving interactions among astrocytes, fibroblasts, pericytes, and immune system cells. Perivascular cells migrate into the injury zone and participate in forming the fibrotic component, which reinforces the mechanical density of the scar. These processes are regulated by the JAK/STAT, TGF-β, and NF-κB signaling pathways, which control inflammation and cellular proliferation [86].

### 2.3. Microglia and Neuroinflammation

Microglial cells, as the principal effectors of innate immunity in the CNS, respond rapidly to SCI [87]. The destruction of neurons, axons, glial cells, and the vascular bed is accompanied by the release of DAMPs [88,89,90]. Recognition of DAMPs by the innate immunity receptors of microglia (TLR2, TLR4, P2X7) initiates a cascade of NF-κB and MAPK signaling pathways that lead to a pronounced morphological, functional, and transcriptional remodeling of microglia, serving as a trigger for the unfolding of a complex cascade of inflammatory and neurodegenerative processes [91].

During the first minutes after injury, resting microglia, which have numerous cytoplasmic processes, do not retract them but instead elongate and reorient them toward the lesion. The mobility and high dynamism of microglial processes are ensured by the action of ATP through P2Y12 receptors [92,93,94], as well as by nitric oxide and the fractalkine receptor CX3CR1 [95,96], which trigger cytoskeletal remodeling and determine the early microglial response to tissue damage.

After roughly 30 min, microglial processes form a dense border around the injury that mechanically isolates the lesion and limits the spread of the damage. Only at later stages (within 1 to 3 days) activated microglia retracts processes and acquire the classic amoeboid form, which allows it to migrate to the injury site and carry out phagocytosis [97,98].

During the first 24 h following traumatic spinal cord injury (TSCI), microglia dominate the injury zone, releasing pro-inflammatory cytokines and chemokines. Under the influence of reactive oxygen species (ROS) and elevated intracellular calcium, the NLRP3 inflammasome is activated, facilitating the processing and secretion of IL-1β and IL-18. By days 1 to 3, a significant subset of the microglial population begins to express molecules associated with reparative and neuroprotective functions—such as IL-10, TGF-β, and IGF-1—alongside pro-inflammatory factors, which contributes to the attenuation of oxidative stress and the stimulation of regenerative processes. However, under persistent intense inflammation, microglia exhibit enhanced production of pro-inflammatory cytokines (TNF-α, IL-1β, IL-6), chemokines, and nitric oxide (NO•). NO• reacts with superoxide anion (O_2_^−^•) to form peroxynitrite (ONOO^−^)—a potent oxidant and nitrating agent [99,100,101]. ONOO^−^ induces lipid peroxidation, mitochondrial dysfunction, energy deficit, and protein nitration [102,103,104,105], thereby exacerbating neurotoxicity and expanding the zone of secondary damage. Sustained pro-inflammatory microglial activation contributes to the chronification of neuroinflammation, impaired oligodendrogenesis, and defective axonal regeneration. It is important to emphasize that, under conditions of traumatic CNS injury, the functional phenotype of microglia does not conform to a binary classification (pro-inflammatory versus anti-inflammatory); rather, a dynamic continuum is observed, in which an individual cell may acquire properties associated with both inflammation and regeneration/neuroplasticity, depending on the balance of microenvironmental signals, the time elapsed since injury, and the distance from the lesion epicenter.

Substantial CNS damage is characterized by inefficient clearance of cellular debris and protracted inflammation [90]. A key mechanism delaying recovery is the slow phagocytosis carried out by myeloid cells. In rodents, the temporal dynamics of phagocytic activity are reflected by CD68 expression: a low level in the first days after injury gives way to a peak on day 4, after which, from days 14 to 35, microglia reduce CD68 and increase the homeostatic marker P2RY12, signaling a return to the resting phenotype. However, even in rodents the complete removal of myelin remnants takes weeks and months, and in humans it sometimes takes years [106,107], which leads to persistent presence of cellular debris and myelin bodies that block axonal regeneration and oligodendrogenesis. An important regulator linking phagocytosis with the anti-inflammatory response is the receptor TREM2 (triggering receptor expressed on myeloid cells-2), expressed on microglia. Activation of TREM2 enhances the engulfment of apoptotic cell remnants and suppresses the secretion of pro-inflammatory cytokines, whereas its dysfunction leads to accumulation of debris and overproduction of TNF-α/IL-1β [108].

## 3. Serotonergic System Before and After SCI

### 3.1. Sources of Serotonin in the Spinal Cord

The main source of serotonin in the CNS is the raphe nuclei, located in the brainstem [109,110]. Descending serotonergic projections to the spinal cord play an important role in modulating the sensorimotor and autonomic functions that are substantially disrupted in SCI.

Descending serotonergic pathways, whose fibers originate from the raphe nuclei in the reticular formation of the brainstem, form part of the reticulospinal and bulbospinal tracts [111]. The reticulospinal tract participates in maintaining muscle tone, balance, and posture, as well as in coordinating movements and ensuring postural stability; in addition, it is involved in autonomic processes [112]. The bulbospinal projections control movements and their coordination [113] and also participate in regulating cardiovascular activity, respiration, urination, defecation, and vascular tone [111]. In both pathways, serotonin increases the excitability of motoneurons [114] and exerts an analgesic effect [115]. Unlike the tracts listed above, the ventral corticospinal tract is not serotonergic; however, serotonin released in the spinal cord can modulate its activity by influencing motoneuron excitability and the suppression of pain transmission [116]. The ventral corticospinal tract plays a key role in controlling voluntary movements, especially movements of the trunk and proximal limb segments, as well as in maintaining posture and muscle tone [116].

After SCI, a reduction in serotonin levels is observed not only in the spinal cord but also in the brain. This is related to a decrease in afferent feedback from lower segments, which leads to reduced activity of the raphe nuclei [117]. Additional suppressive influences come from stress, chronic pain, and the inflammatory processes that accompany the injury [118]. Because the dorsal raphe nuclei project onto structures of the limbic system, disturbances in their function are also reflected in the psychoemotional sphere, raising the risk of affective disorders (primarily depression and anxiety) [118] as well as cognitive impairments [119]. At the same time, serotonergic neurons possess a high capacity for recovery: their axons sprout and reorganize relatively quickly after injury [120], which makes the serotonergic system a promising target for therapeutic modulation of neuroplasticity and regeneration [5].

There are data indicating that after SCI cells containing the enzyme aromatic L-amino acid decarboxylase (AADC) are able to synthesize serotonin from its precursor, 5-hydroxytryptophan. This occurs as a consequence of the loss of descending serotonergic projections, which causes a phenotypic change in AADC cells that allows them to produce serotonin locally. Such local serotonin production by AADC cells can increase the excitability of spinal motor neurons, contributing to phenomena such as the hyperreflexia observed below the level of injury [121].

Peripheral sources of serotonin, such as platelets, adipose cells, and immune cells, also participate in regulating spinal cord function. Platelets, being the main peripheral source of serotonin, take it up from the bloodstream and store it in dense granules. The release of serotonin from platelets can affect vascular tone (vasodilation upon binding to 5-HT2 receptors and vasoconstriction upon binding to receptors of other subtypes) and blood flow [122], which indirectly influences spinal cord function by regulating the supply of nutrients and oxygen. Serotonin released by platelets may also participate in regulating immune responses, which can affect the overall state of the spinal cord and its recovery processes [123]. The presence of serotonin in hematopoietic and immunocompetent tissues points to its role in the local regulation of hematopoiesis and immunogenesis, which may influence the mechanisms of spinal cord recovery [124].

Adipose tissue is another peripheral source of serotonin, although its role in the spinal cord is less direct than that of other sources. Serotonin in adipose cells participates in metabolic processes, including carbohydrate metabolism and energy balance, which can affect the overall health of the organism and indirectly influence spinal cord function [122].

### 3.2. Dynamics of Serotonin Levels After Injury

In the first minutes following SCI, a massive release of serotonin (5-HT) occurs, driven by parallel mechanisms. First, serotonin is released from platelets at the site of vascular rupture. Platelets release it upon activation by coagulation factors, ATP, and collagen following disruption of the subendothelial membrane [125]. The released serotonin binds to 5-HT_2A_ receptors on vascular smooth muscle cells, inducing vasoconstriction, and also participates in platelet aggregation, which is crucial for limiting blood loss [125,126]. Second, the destruction of descending serotonergic axons originating in the brainstem raphe nuclei results in a massive release of neuro [125,126] genic serotonin directly into the spinal cord parenchyma. This has been demonstrated in animal experiments, where serotonin concentrations in the spinal cord rostral to the injury site can increase approximately twofold compared to controls [127]. Such an acute serotonergic surge is accompanied by increased neuronal network excitability and may promote the generation of rhythmic motor activity. Similar effects have been observed in humans: administration of the serotonin precursor 5-hydroxytryptophan (5-HTP) to patients with chronic SCI enhances cutaneous-muscular reflexes and increases muscle activity during passively induced movements [128].

These two sources lead to a sharp elevation in 5-HT levels, with concentrations in the injury zone potentially rising tenfold or more above baseline within the first 30 min. However, this acute surge has conflicting consequences. On the one hand, platelet-derived serotonin provides vascular protection by limiting hemorrhage [125]. On the other hand, excessive vasoconstriction mediated by 5-HT_2A_ receptors exacerbates local ischemia and promotes microvascular thrombosis, thereby expanding the zone of secondary damage. Furthermore, elevated serotonin levels in the acute phase correlate with increased expression of c-fos—a marker of the early injury response—whose prolonged activation is considered a predictor of neuronal apoptotic death [129,130]. The highest c-fos expression is detected in the area of perifocal edema following SCI. In serotonin-deficient rats with SCI, edema and c-fos immunoreactivity were not observed. Administration of para-chlorophenylalanine (a selective inhibitor of tryptophan hydroxylase) to injured animals led to a significant reduction in c-fos expression. These findings suggest that the massive serotonin release resulting from the destruction of 5-HT-containing fibers and platelet release can initiate a cascade of molecular events mediating neuronal apoptosis and may also contribute to the development of post-traumatic edema [131].

In chronic pain, serotonin release in the spinal cord may exert pronociceptive effects. In models of chronic pain, enhanced tonic—rather than phasic—serotonergic signaling has been linked to increased nociception, suggesting that under certain conditions, the role of serotonin may shift from modulatory to exacerbating [132].

As the condition transitions to the chronic stage, dysfunction of descending serotonergic projections originating from the raphe nuclei leads to serotonin deficiency below the level of injury [112]. In response, compensatory hypersensitivity of serotonin receptors and changes in their expression develop, contributing to spasticity and other motor impairments. These receptor and synaptic rearrangements reflect broader neuroplastic processes and play a dual role: on the one hand, they underlie pathological states; on the other, they may facilitate partial functional recovery after SCI [132]. Chronic serotonin deficiency has prompted investigation into therapeutic strategies, such as the use of serotonergic agents and receptor agonists, aimed at restoring motor function and alleviating symptoms including spasticity and pain [5]. Modulation of serotonergic pathways represents a promising approach to accelerating recovery after SCI. Pharmacological interventions, including the use of 5-HTP and serotonin receptor agonists, have demonstrated potential in improving motor function and reducing pathological symptoms [133]. The ability of the serotonergic system to adapt and regenerate following injury is a key endogenous factor promoting recovery. Strategies that stimulate the growth of new serotonergic branches and modulate receptor activity may contribute to functional restoration and improved rehabilitation outcomes after SCI [5].

### 3.3. Overview of Serotonin Receptors (5-HTR) in the CNS

There are seven types of serotonin receptors (5-HT_1_–5-HT_7_) and numerous subtypes, each characterized by a distinct cell-type-specific expression pattern and unique functional features (Table 1). Activation of these receptors modulates neuronal activity and contributes to the diverse effects of serotonin in the CNS, which plays a key role in maintaining its homeostasis. The assignment of serotonergic receptors to one class or another (metabotropic or ionotropic) is based on the mechanisms of intracellular signal transduction [134]. Most 5-HT receptors belong to the class of G-protein-coupled receptors (GPCRs), which transmit signals through G proteins, triggering various intracellular signaling cascades. These include the 5-HT_1_, 5-HT_2_, 5-HT_4_, 5-HT_5_, 5-HT_6_, and 5-HT_7_ receptors [135]. The binding of serotonin to these receptors leads to activation of the corresponding G proteins (for example, G_i,_ G_s_, G_q_), which in turn regulate effector proteins such as adenylyl cyclase, phospholipase C, and ion channels, thereby changing the levels of second messengers.

5-HT_1_ receptors are generally coupled to G_i_ proteins, resulting in inhibition of adenylyl cyclase and a decrease in intracellular cAMP levels [136]. Reduced cAMP levels are associated with diminished neuronal excitability and firing frequency, which can modulate behavioral and cognitive functions. In addition, βγ subunits released from G_i/o_-proteins heterotrimers can initiate alternative signaling cascades, including activation of phospholipase C (PLC), MAPK/ERK, and PI_3_K-Akt [137,138].

The 5-HT_2A_ receptor is predominantly coupled to G_q/11_ proteins, leading to phospholipase C (PLC) activation and intracellular calcium mobilization. This G_q/11_-mediated PLC activation is the key signaling mechanism underlying the psychoactive effects of serotonergic compounds; its inhibition attenuates the psychedelic-induced head-twitch response in animals [138]. β-Arrestins are involved in receptor desensitization and internalization, as well as in signaling through alternative pathways. In particular, β-arrestin-2 is associated with suppression of receptor activity and tachyphylaxis, which may influence the therapeutic properties and side-effect profile of drugs acting at the 5-HT_2A_ receptor [139].

Like the 5-HT_2A_ receptor, the 5-HT_2C_ receptor transduces signals predominantly via the G_q/11_ pathway, leading to activation of phospholipase C (PLC) and subsequent intracellular calcium mobilization. The 5-HT_2C_ receptor exhibits a broad coupling spectrum, interacting with G_i/o/q_ and G_12/13_ proteins. This feature contributes to its diverse physiological functions and potential therapeutic applications. The 5-HT_2C_ receptor preferentially binds β-arrestin-2 over β-arrestin-1, which may influence the dynamics of receptor desensitization and internalization [139].

The 5-HT_3_ receptor is unique among serotonin receptors in that it functions as a ligand-gated ion channel, forming homo- or heteropentamers like nicotinic receptors [140]. 5-HT_3_ receptors are ligand-gated ion channels that, upon activation, permit influx of cations, particularly Na^+^, K^+^, and Ca^2+^, leading to rapid neuronal depolarization and subsequent neurotransmitter release. 5-HT_3_ receptors are expressed in brain regions associated with anxiety, such as the medial prefrontal cortex, amygdala, and hippocampus, as well as in the spinal cord.

5-HT_4_ receptors signal via the G_s_/cAMP/PKA pathway, regulating neurotransmitter release and synaptic plasticity. Additionally, 5-HT_4_ activates the ERK pathway via Src tyrosine kinase in a β-arrestin-independent manner, contributing to cognitive processes and memory formation [141]. In the hippocampus, 5-HT4 receptor stimulation enhances locomotor activity, an effect blocked by 5-HT_4_ antagonists, indicating their modulatory role in locomotion [142].

5-HT_5A_ receptors play an important role in regulating astrocytic function and possibly in suppressing reactive astrocytic responses, which is relevant in conditions such as gliosis [143]. In addition to the classical G_i_/G_o_-mediated pathway, these receptors inhibit ADP-ribosyl cyclase, affecting the formation of cyclic ADP-ribose, indicating a unique coupling mechanism that may influence calcium signaling and other cellular processes [144]. Furthermore, activation of 5-HT_5A_ receptors can induce transient opening of potassium channels, likely due to an increase in intracellular calcium following inositol trisphosphate formation. This effect is inhibited by heparin and pertussis toxin, suggesting crosstalk between G_i_/G_o_ proteins and the phospholipase C cascade [144].

The 5-HT_6_ receptor is predominantly coupled to G_s_ protein, stimulating adenylyl cyclase and triggering classical cAMP-dependent signaling. This pathway mediates the receptor’s involvement in the regulation of neurotransmitter systems and cognitive functions [145]. In addition, the 5-HT_6_ receptor modulates the activity of Cdk5, a kinase critically important for neuronal migration and neurite outgrowth during nervous system development [146]. Moreover, this receptor influences mTOR and Fyn-kinase signaling cascades, which are involved in synaptic plasticity and cognitive processes [147]. An important aspect is the interaction of 5-HT_6_ with microtubule-associated protein 1B light chain 1 (MAP1B-LC1), which regulates intracellular receptor trafficking and cell-surface expression [148].

Activation of the 5-HT_7_ receptor in most cases activates the G_s_-protein–adenylyl cyclase (AC) pathway. The latter mediates such physiological effects as serotonin-induced phase shifts in circadian rhythms, modulation of memory and motor activity, and is responsible for the coordinated rhythmic activity of neurons governing the movement of right and left limbs during locomotion [149]. In spinal locomotor mechanisms, 5-HT_7_ receptors, together with 5-HT_1A_, participate in the regulation of rhythmogenesis: their antagonists reduce step-cycle duration, consistent with their action on neurons responsible for the rhythmogenic function of the central pattern generator (CPG) [150]. In contrast to 5-HT_2_A receptors, 5-HT_7_ receptors do not directly affect motoneurons but act primarily at the level of CPG interneurons, facilitating the activity of the rhythm generator itself [151]. Furthermore, the 5-HT_7_ receptor can couple to G_12_ protein, activating small Rho-family GTPases, which are involved in cytoskeletal reorganization and neurite outgrowth [152]. It can also signal through β-arrestin pathways independently of G-protein activation. This pathway is thought to participate in ERK activation, which plays a key role in pain modulation. Activation of 5-HT_7_ stimulates neurite elongation via pathways involving mTOR, Cdc42, and actin filament dynamics. This process is crucial for neural development and the formation of neuronal connections, underscoring the receptor’s role in shaping neuronal morphology [153].

**Table 1:** Serotonin receptor subtypes in the CNS: a summary pathway and associated functional effects.

Receptor	Signaling	Effects
5-HT_1_	G_α/i/o_ → PLC → PKC, ↑ Ca^2+^	Neuritogenesis/synaptogenesis [137]
	G_α/i/o_ → ↓ cAMP; MAPK/ERK; PI_3_K/Akt;	5-HT_1A_—generation of rhythmic motor activity; neuronal development via cytoskeletal modulation through MAPK and PI3K–Akt signaling pathways [137,151] 5-HT_1B_—modulation of synaptic plasticity and emotional behavior via ERK_1/2_ phosphorylation [154] 5-HT_1E_—neuron survival; regulation of cell cycle and survival-associated genes (cMyc, cyclin D1, BCL2) [155]
5-HT_2_	G_q/11_ → PLC → IP_3_/DAG → ↑ Ca^2+^, PKC;	Psychoactive effects mediator; G_q_ signaling inhibition reduces head-twitch response to psychedelics in animals [138,156]
	Activate PLD via ARF-dependent	5-HT_2A_—enhancement of motoneuron and premotor interneuron excitability [150,151]; amplitude modulation of locomotor patterns; blockade reduces ventral root activity amplitude [150]. Key role in spinal locomotor generator–to–executive circuit transmission
5-HT_3_	Ligand-gated ion channel (Na^+^/K^+^/Ca^2+^ influx) → depolarization	Motor control: activation in VTA enhances locomotor activity via mesolimbic dopaminergic system modulation [157]; induction of rhythmic locomotor-like hindlimb movements after spinal cord injury, indicating potential role in spinal locomotor generation [158]
5-HT_4_	Src/ERK (β-arrestin-independent)	Modulation of cognitive processes and memory formation [141]
	G_s_ → ↑ cAMP → PKA;	Regulation of neurotransmitter release and synaptic plasticity, particularly in learning and memory; hippocampal stimulation enhances motor activity in rats (antagonist-sensitive), indicating modulatory role in locomotion [142]
5-HT_5A_	G_i/o_ → ↓ cAMP; inhibits ADP-ribosyl cyclase; regulates K^+^ channels via PLC/IP_3_	Role in the suppression of reactive astrocytic responses [143]
5-HT_6_	G_s_ → ↑ cAMP → PKA; Cdk5; mTOR; Fyn-kinase; MAP1B-LC1 interaction	Regulation of neurotransmitter systems and cognitive functions [145]; modulation of Cdk5 (neuronal migration, neurite outgrowth) [146]; influence on mTOR and Fyn kinase cascades (synaptic plasticity, cognition) [147]; interaction with MAP1B-LC1 regulating receptor trafficking and surface expression [148]
5-HT_7_	G_s_ → ↑ cAMP → PKA;	Serotonin-induced circadian rhythm phase shifts; memory and motor activity modulation; coordination of rhythmic neuronal activity for left/right limb movements during locomotion [149]; rhythmogenesis regulation [150]. Stimulation of neurite elongation via mTOR, Cdc42, and actin filament dynamics [153]
	G_12_ → Rho GTPases	Cytoskeletal reorganization and neurite outgrowth [152]
	β-arrestin → ERK	Involvement in pain modulation

**↑ increase; ↓ decrease; → leads to**.

## 4. Serotonergic Modulation of Glial Responses After SCI

### 4.1. Serotonin and Oligodendrocytes

Serotonin (5-HT) is one of the earliest-appearing pleiotropic neurotransmitters of the CNS, participating in the regulation of neurogenesis and synaptic plasticity [159]; however, its influence on cells of the oligodendroglial lineage has been studied very little. The available information is largely limited to the period of embryonic development or to in vitro studies, while experiments on adult animals under normal and pathological conditions have essentially not been carried out. Direct experimental data convincingly demonstrating an influence of serotonergic transmission on OPC proliferation and maturation, as well as on the processes of de novo myelination and remyelination, have in fact not been obtained to date.

Cells of the oligodendroglial lineage express 5-HT_1_A and 5-HT_2_A receptors, and the level of their expression changes during differentiation [160]. According to immunohistochemical analysis, immunoreactivity for 5-HT_1_A and 5-HT_2_A is weakly detected at the stage of early precursors (NG_2_+) but increases considerably during maturation, reaching a maximum at the stage of myelinating oligodendrocytes (MBP+) [160]. At the same time, in primary OPC cultures the 5-HT_2_A subtype is predominantly expressed, which suggests that under certain conditions or depending on the developmental stage, the dominance of one serotonergic receptor subtype may be decisive for the regulation of OPC function and their subsequent activity [161].

In primary cultures of OPCs isolated from rat cortex, chronic exposure to 5-HT led to suppression of differentiation, reduced myelination, and induction of apoptotic death of oligodendroglial-lineage cells. Sensitivity to 5-HT depended on the developmental stage: immature oligodendrocytes were the most vulnerable to 5-HT. Their treatment with 5-HT caused a dose-dependent morphological rearrangement, manifested in a reduction in the number and length of processes and in shortening of the internodal length. Immunoreactivity for the differentiating-cell marker GalC was confined to the perikaryon and was practically undetectable in the processes, whereas in control cells the staining was distributed evenly across the entire surface. Similarly, cells positive for the mature-oligodendrocyte marker Rip displayed fewer and deformed processes, which contrasted sharply with the extensive branched network in the controls. Quantitative analysis confirmed a significant reduction in the surface area of oligodendrocytes treated with 5-hydroxytryptophan (5-HTP) compared with control cells. At the molecular level, exposure to 5-HT led to reduced expression of the major myelin proteins MBP and PLP, as well as to decreased activity of the enzyme CNPase in cultures of immature oligodendrocytes. Notably, apoptosis was observed in immature oligodendrocytes, whereas OPCs remained viable even at high concentrations of 5-HT (up to 250 µM). It is proposed that this apoptotic effect is mediated by 5-HT_2_A receptors [160]. Thus, the available data indicate that serotonin is able to inhibit maturation and cause the death of immature oligodendrocytes, potentially impeding remyelination. However, all of these results were obtained under normal conditions. It remains unclear whether this vulnerability persists under conditions of an inflammatory microenvironment, for example in neurotrauma.

At the same time, a study on the immortalized oligodendrocyte line M03–13 demonstrated an opposite role of serotonin: 5-HT activates PLC/PKC/ROS-dependent signaling cascades, stimulating OPC maturation and enhancing antioxidant defense and mitochondrial function, which is a critical factor for ensuring myelination. In particular, treatment with 5-HT increased the expression of the mature-oligodendrocyte markers MBP and Olig2. The authors showed that this effect is mediated by NOX-dependent generation of ROS, since the use of NOX inhibitors completely abolished the observed changes. Moreover, inhibition of cell migration correlated with an increase in ROS levels, which in turn modulate the state of the cytoskeleton [161]. Functionally active mitochondria are necessary to maintain the normal state of oligodendrocytes, which are characterized by a high level of metabolism and vulnerability to hypoxia [162]. The process of oligodendrocyte differentiation requires a metabolic shift toward increased energy production, which is accompanied by increased expression of several genes associated with mitochondrial β-oxidation (*Echs1*, *Hadhsc*, *Hadh2*, *Hadha*). The myelin sheath consists of more than half of lipids synthesized by oligodendrocytes [163,164,165]. The biosynthesis of cholesterol, critically necessary for myelin formation, requires a large amount of acetyl-CoA produced in mitochondria during the β-oxidation of fatty acids [166,167,168]. The use of a mitochondrial uncoupler showed that the energy deficit it caused activates protein phosphatases and leads to selective inactivation of MBP-associated protein kinases, making both the maintenance and the initiation of myelination impossible [169]. Because cholesterol biosynthesis is energy-intensive, under conditions of oxygen deprivation in SCI the immature oligodendrocytes that embark on the path of differentiation into mature myelinating oligodendrocytes prove to be the most vulnerable [170]. A deficit of the energy needed to support the processes of transition into mature oligodendrocytes ultimately leads to their death and to the failure of reparative remyelination. Notably, although abundant data indicate that OPCs proliferate under these conditions [160], their maturation into mature oligodendrocytes proceeds extremely slowly and inefficiently [171,172]. The reasons for this may be related both to the inflammatory microenvironment and to insufficient energy supply of immature oligodendrocytes. In this connection, the finding that 5-HT enhances mitochondrial function and promotes energy production in immature oligodendrocytes through the PLC/PKC/ROS axis opens a promising avenue for the use of serotonergic agents starting from the second week after SCI (the onset of differentiation) [10].

The data presented for the immortalized oligodendrocyte line M03–13 come into obvious conflict with the results obtained in primary cultures. The most likely explanation for this discrepancy lies in differences between the experimental models. The M03–13 line, unlike primary oligodendrocytes, expresses exclusively the 5-HT_2_A receptor subtype and does not contain 5-HT_1_A [161], which may fundamentally alter the balance of intracellular signaling. In addition, immortalized cells have different proliferative and metabolic characteristics [173], which potentially affects their sensitivity to serotonergic stimulation. Consequently, the interpretation of 5-HT effects requires strict consideration of the cell model, and the universality of the observed phenomena remains contentious.

An interesting observation was obtained in co-cultures of oligodendrocytes and neurons. Under these conditions, 5-HT did not lead to oligodendrocyte death or to a decrease in their density. However, at the sites of the nodes of Ranvier, an abnormal clustering of the contact-associated protein (Caspr) was observed, which suggests that exposure to 5-HT disrupts the axon-dependent regulation of myelination, thereby suppressing formation of the myelin sheath: the Caspr protein is necessary for anchoring the myelin sheath to the axonal membrane. Caspr is expressed diffusely on the axon surface before the onset of active myelination and, upon completion of myelination, gradually concentrates in the paranodal domain (the region of the myelinated fiber located on both sides of the node of Ranvier) [174]. Caspr clustering was chaotic and irregular in cultures of oligodendrocytes treated with 5-HT relative to the control group, where Caspr was located at equal distances from one another.

Additional evidence of a protective role of serotonin was obtained in an in vitro study in which paroxetine (an SSRI) significantly increased the viability of primary oligodendrocytes and also enhanced their proliferation and maturation [175]. In the same work, in a mouse model of depression induced by chronic corticosterone administration, paroxetine (an SSRI) stimulated OPC proliferation (Olig2+/NG_2_+) and oligodendrocyte differentiation (Olig2+/CC-1+), increased the thickness of the myelin sheath, and restored its integrity in the prefrontal cortex. These data are consistent with the effects described for the M03–13 line, but they were obtained under conditions of systemic administration, which does not rule out indirect mechanisms. Interestingly, in vivo studies also contain indirect data on a negative influence of serotonin on the normal development of oligodendrocytes. For example, administration of SSRI-class drugs to pregnant rats causes not only disruption of cortical neuronal connections in the fetus but also oligodendrocyte pathology, demyelination, and social behavior resembling autism spectrum disorders [176].

**Figure 2:**
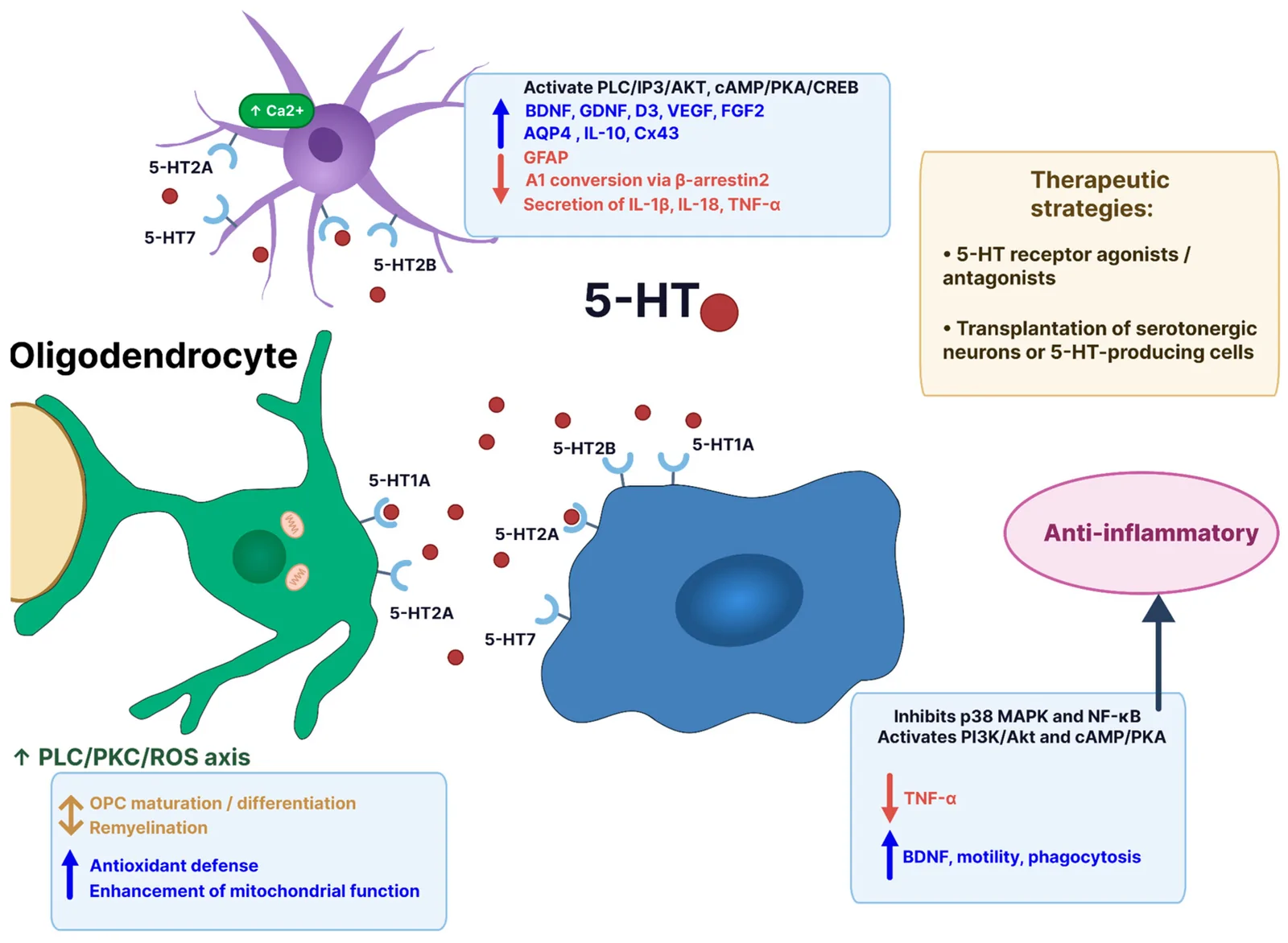
Influence of 5-HT on astrocytes, microglia, and oligodendrocytes. IL1β–Interleukin-1 beta, TNF-α–Tumor Necrosis Factor alpha, BDNF–Brain-Derived Neurotrophic Factor, GDNF–Glial cell line-Derived Neurotrophic Factor, D3–Deiodinase 3, VEGF–Vascular Endothelial Growth Factor, FGF2–Fibroblast Growth Factor 2, AQP4–Aquaporin-4, IL-10–Interleukin-10, CX43–Connexin-43, PLC/PKC/ROS–Phospholipase C/Protein Kinase C/Reactive Oxygen Species, PLC/IP_3_/AKT–Phospholipase C/Inositol 1,4,5-Trisphosphate/Protein Kinase B (PKB), cAMP/PKA/CREB–Cyclic Adenosine Monophosphate/Protein Kinase A/cAMP Response Element-Binding Protein, NF-kB–Nuclear Factor kappa-B, p38 MAPK–p38 Mitogen-Activated Protein Kinase.

Thus, the literature has accumulated contradictory data on the influence of 5-HT on cells of the oligodendroglial lineage. In a number of studies, serotonergic stimulation suppressed oligodendrocyte differentiation and, accordingly, myelination, whereas in others it activated them instead. These divergent effects are probably determined by the developmental stage of the oligodendrocytes, the microenvironment, the experimental conditions, and the type of predominant serotonergic receptors. To date, studies on the influence of 5-HT on remyelination processes in SCI are practically absent. At the same time, serotonergic modulation has already demonstrated improvement of sensorimotor recovery after SCI. It may be assumed that, under conditions of neuroinflammation, the serotonergic system, directly or indirectly, exerts a favorable influence on the state of oligodendroglial-lineage cells (Figure 2).

It is necessary to draw an important methodological distinction between data obtained using 5-HT, selective agonists/antagonists of 5-HT receptors, and data obtained in studies using selective serotonin reuptake inhibitors (SSRIs). This distinction is equally relevant for the following sections, which discuss serotonergic modulation of astrocytes and microglia. The direct use of serotonin or its receptor-specific ligands makes it possible to unequivocally link the observed effects to the activation or blockade of a specific subtype of 5-HT receptors on glial cells. In contrast, SSRIs (e.g., fluoxetine, paroxetine) act by inhibiting serotonin reuptake, thereby increasing the concentration of endogenous serotonin in the synaptic cleft and the extracellular space. It is important to note that this mechanism of action is fully effective only in in vivo systems where serotonergic neurons or other cells producing serotonin are present. However, in the in vitro systems considered in this review, namely, cultures of oligodendrocytes, astrocytes, and microglia, serotonin is not synthesized de novo. Consequently, the classic mechanism of action of SSRIs (inhibition of serotonin reuptake) is not applicable here. Instead, it was shown that some SSRIs act as direct agonists of certain subtypes of serotonin receptors, since the effect of SSRIs disappeared when the activated 5-HT receptor was blocked. Thus, in the context of isolated cell cultures, SSRIs primarily serve as pharmacological agents for the direct activation of serotonergic receptors, thereby mimicking the action of serotonin. Nevertheless, it is important to recognize that SSRIs may engage additional, as yet unidentified molecular pathways and cross-interactions in vitro, which may also contribute to the observed effects. In vivo, the action of SSRIs is even more complex, as it involves both an increase in extracellular serotonin levels and direct receptor-mediated effects on various cell types, including neurons and glia, so such results should be interpreted with caution. Nevertheless, SSRIs provide valuable information about the potential therapeutic effects of increasing serotonergic tone.

### 4.2. Serotonin and Astrocytes: The Role of 5-HT in Reactive Astrogliosis

Astrocytes express several types of serotonergic receptors: 5-HT_1A-F_, 5-HT_2A-2C_, 5-HT_5A_, 5-HT_6_, and 5-HT_7_ [143,177,178,179,180,181], which participate both in ensuring normal function and in the development of pathological processes in the CNS [180,181,182,183].

By acting on the receptors listed above, serotonin is able to direct the phenotypic polarization of astrocytes through the activation of intracellular signaling cascades. In particular, stimulation of the 5-HT_2B_ receptor triggers the PI_3_K/AKT and MAPK/ERK pathways. Activation of the MAPK/ERK cascade leads to changes in the expression of many functionally significant proteins [184]. These include Ca^2+^-dependent phospholipase A2 (cPLA2 IVA), the kainate receptor GluK2, the canonical TRP channel TRPC1, the L-type voltage-gated Ca^2+^ channel Cav1.2, the enzyme ADAR2 (adenosine deaminase acting on RNA), and the structural protein caveolin-1 (Cav-1) [180,184,185,186,187,188]. It is important to emphasize that cPLA2 IVA plays a neuroprotective role owing to the mobilization of arachidonic acid, a precursor of prostaglandins and other pro-inflammatory mediators. This in turn leads to suppression of the production and secretion of a number of pro-inflammatory molecules [189], which represents a protective mechanism limiting inflammation in the CNS.

With respect to Cav-1, it has been established that chronic stimulation of 5-HT_2B_ receptors with low doses of fluoxetine which acts with high affinity at the 5-HT_2B_ receptors causes a decrease in the expression of this protein, which correlates with a reduction in the functional activity of excitatory amino acid transporters (EAATs) and, accordingly, with a decrease in glutamate uptake from the extracellular space. Presumably, this effect is mediated by disruption of the stable incorporation and anchoring of EAATs in the plasma membrane owing to a deficit of Cav-1 [190]. From this it may be inferred that, under conditions of neurotrauma, prolonged administration of 5-HT_2B_ receptor agonists would enhance excitotoxicity through the induced reduction in Cav-1 expression and the subsequent disruption of astrocytic glutamate uptake; therefore, when using fluoxetine to improve recovery after injury, its dose-dependent effect on the glutamate uptake system must be taken into account.

It has also been demonstrated that the indirect action of SSRIs on serotonergic receptors stimulates the production of neurotrophic factors in vitro. For example, in experiments on primary astrocyte cultures, chronic fluoxetine administration led to increased expression of brain-derived neurotrophic factor (BDNF) and its receptors, glial cell line-derived neurotrophic factor (GDNF), deiodinase 3 (DIO3), vascular endothelial growth factor (VEGF), and fibroblast growth factor 2 (FGF2) [191,192,193]. This effect is mediated by CREB-dependent transcription activated through cAMP/PKA signaling as well as through the MAPK/ERK cascade [194]. Notably, SSRIs (fluoxetine and paroxetine) increased the mRNA levels of *Bdnf*, *Vegf*, and *Vgf* in cultured astrocytes, whereas tricyclic antidepressants (desipramine and imipramine) had no such effect [195]. Thus, selective activation of 5-HT_2B_ receptors may serve as a strategy for stimulating the release of growth factors and creating a more favorable microenvironment under SCI conditions.

In addition to its trophic influence, fluoxetine exhibits a pronounced anti-inflammatory effect on astrocytes. In vivo and in vitro studies have shown that it blocks the pro-inflammatory transformation of astrocytes through a non-canonical, β-arrestin-2-dependent mechanism coupled to 5-HT_2B_ receptors, thereby providing neuroprotection [183]. In addition, fluoxetine-mediated activation of 5-HT_2B_ inhibits assembly of the NLRP3 inflammasome [196], which prevents the processing of the pro-inflammatory cytokines IL-1β and IL-18 into their bioactive forms [197]. The consequence of this is a reduction in the overall inflammatory background and a decrease in astrocyte reactivity [183]. Taken together, these data point to a dual mechanism of action of fluoxetine (trophic and anti-inflammatory), which supports its potential therapeutic value in neuroinflammation.

In addition to fluoxetine, another member of the SSRI class, sertraline, also induced an anti-inflammatory phenotype in astrocyte cultures. This was manifested in increased secretion of IL-10 and suppression of TNF-α production under conditions of LPS-induced inflammation. Similar anti-inflammatory effects were also recorded for tricyclic antidepressants. In particular, clomipramine and imipramine reduced the production of nitric oxide and TNF-α [198]; imipramine additionally attenuated the TNF-α-mediated inflammatory response [199], whereas amitriptyline and nortriptyline suppressed the secretion of IL-1β and TNF-α in mixed glial cultures [200]. Thus, both SSRIs and tricyclic antidepressants (TCAs) demonstrate pronounced anti-inflammatory activity toward astrocytes, which broadens the potential for their therapeutic use in controlling secondary inflammation in neurotrauma.

Importantly, there is a reciprocal relationship between inflammation and the serotonergic system. Pro-inflammatory cytokines (IL-1β, TNF-α, and IL-6) participate in the regulation of serotonin homeostasis in vivo, modulating the activity and metabolism of 5-HT in the CNS [201,202]. One of the key mechanisms is enhanced transcription of the *SERT* gene, which encodes the high-affinity serotonin reuptake transporter. In particular, prolonged exposure to TNF-α significantly increases the expression and functional activity of SERT in primary astrocytes [203]. This is confirmed by experiments in which treatment of astrocytes with TNF-α led to a sustained increase in SERT-mediated 5-HT uptake by the cells. Under conditions of chronic inflammation, such an increase in serotonin transporter (SERT) activity can substantially lower the concentration of extracellular serotonin. Moreover, astrocytes rapidly degrade the captured 5-HT because they express large amounts of monoamine oxidase A (MAO A) [204]. The combination of these processes can lead to a significant reduction in the availability of serotonin for neuronal and glial receptors and, consequently, contribute to the development of the serotonergic dysfunction characteristic of neuroinflammatory disorders.

SSRIs have also been shown to affect intercellular communication among astrocytes through gap junctions. For example, fluoxetine and duloxetine increase the expression of connexin 43 (Cx43), the main gap-junction protein in astrocytes. This effect prevents the stress-induced decrease in gap-junction permeability, thereby maintaining the functional integrity of the astrocytic network [205]. Under pathological conditions, by contrast, gap-junction permeability in the astrocytic syncytium often increases, which promotes the uncontrolled spread of cytotoxic metabolites (K^+^ ions, glutamate, ATP/ADP) across the astrocyte network and the expansion of the secondary-damage zone, for example in ischemia or inflammation [206]. A pharmacologically induced increase in Cx43 expression can offset the pathological rise in permeability, limiting the spread of toxic signals and reducing the involvement of astrocytes located distal to the lesion in reactive astrogliosis. Thus, modulation of Cx43 expression is regarded as an additional mechanism of the neuroprotective action of serotonergic modulation (Figure 2).

Despite convincing evidence of a protective influence of serotonergic modulation on astrocytes, most such studies have been performed in the context of depressive disorders rather than neurotrauma. Studies that directly analyze the role of serotonergic modulation of the reactive astroglial phenotype in SCI are very scarce. Indirect indications of such a connection nevertheless exist: it is known that regeneration of serotonergic axons in the injury zone is accompanied by pronounced astrogliosis [6], which suggests the existence of cross-talk signaling interactions. Nevertheless, direct evidence of the specific mechanisms mediating the influence of the serotonergic system on the phenotype of reactive astrocytes in SCI has not yet been found. In a pharmacological screen of compounds modulating the severity of astrogliosis as a marker of the pathological process, the tricyclic antidepressant clomipramine was shown to reduce GFAP expression in mice by 50% [207]. This observation is a weighty argument in favor of the possibility of pharmacologically modulating the serotonergic system to attenuate excessive astrogliosis. Given that, in the late subacute and chronic phases of injury, astrogliosis contributes to the formation of the glial scar that sustains chronic inflammation and impedes axonal sprouting, targeted action on serotonin receptors may become a promising strategy for improving neuroplasticity.

### 4.3. Serotonin and Microglia: Influence on Microglial Polarization

In line with current views, there is a bidirectional relationship between the serotonergic system and microglia: serotonergic stimulation induces phenotypic transformation of microglia, while microglia in turn modulate the activity of the serotonergic system [208]. Interestingly, the effect of serotonin is context-dependent. Under normal conditions or at elevated 5-HT concentrations, it can enhance microglial activation, whereas in pathological inflammation serotonin more often displays anti-inflammatory and neuroprotective properties [209]. This is explained by differences in receptor expression, in the local concentration of the mediator, and in the state of the cellular environment.

Microglia releasing pro-inflammatory cytokines (IL-1β, TNF-α) directly affect serotonergic innervation. This influence is realized through changes in SERT activity, modulation of the expression and localization of 5-HT receptors, the dynamics of their signal transduction, and the expression of tryptophan hydroxylase-2 (TPH2), the key enzyme of serotonin synthesis in the CNS. Recent studies show that the release of pro-inflammatory cytokines (TNF-α, IL-1β) increases SERT activity through p38-MAPK-mediated phosphorylation. This leads to enhanced serotonin reuptake and a temporary reduction in its availability to neurons [210]. In addition, it has been established that exposure to pro-inflammatory cytokines suppresses TPH2 expression in neurons [208]. Under conditions of low TPH2 expression and activity, tryptophan is actively metabolized along the kynurenine pathway. Indoleamine 2,3-dioxygenase (IDO), the key enzyme of this pathway, is hyperactivated under the action of pro-inflammatory cytokines and leads to the accumulation of quinolinic acid (QUIN), an NMDA receptor agonist, which contributes to increased excitotoxic damage to cells [211,212]. Thus, the data obtained indicate that pro-inflammatory microglia exacerbate the serotonin deficit both directly (through the production of pro-inflammatory cytokines) and indirectly, through the switching of tryptophan metabolism from serotonin synthesis to the production of neuroactive metabolites of the kynurenine pathway. As a result, a pathological vicious circle forms: neuroinflammation reduces the availability of serotonin for both neurons and glial cells, and the deficit of serotonergic signaling in turn helps sustain the activated pro-inflammatory phenotype of microglia.

At the same time, indirect modulation of the serotonergic system via SSRIs promotes microglial reprogramming toward an anti-inflammatory phenotype. The involvement of this system in the regulation of neuroinflammation is supported by experimental evidence [213]: for instance, fluoxetine suppressed TNF-α and IL-6 release in BV2 microglial cells and primary microglial cultures following LPS stimulation [214]. These anti-inflammatory effects are mediated by activation of serotonin receptors expressed on microglia. Although the receptor repertoire varies depending on the microenvironment and the nature of the pathological process [208], stimulation of the 5-HT1A, 5-HT2A, and 5-HT2B subtypes is associated with reduced secretion of IL-1β and TNF-α [209,215]. Furthermore, 5-HT2A receptor activation is linked to enhanced microglial BDNF expression [215].

Joint activation of both receptor pathways initiates the transition of microglia to an anti-inflammatory/neuroprotective phenotype, which promotes the formation of a favorable microenvironment for the regeneration of nervous tissue. Although these data were obtained in models unrelated to SCI (major depressive disorder, neuropsychiatric disorders), they provide strong grounds to suppose that stimulation of 5-HT_1A_ and 5-HT_2A_ on microglia under SCI conditions may lead to a reduced inflammatory background and create conditions for the survival of neurons and oligodendrocytes, which together may substantially improve the prospects for sensorimotor recovery after SCI.

Another serotonin receptor subtype, 5-HT_2B_, is necessary for the development of microglia in the neonatal period, which subsequently prevents their hyperactivity in adulthood and limits the inflammatory response [216].

5-HT_2B_-mediated modulation of macrophages attenuates mechanical and cold allodynia and reduces the severity of neuroinflammation in the dorsal horns of the spinal cord in models of neuropathic pain [217]. Taken together, these data emphasize that the therapeutic effect of SSRIs may be related not only to the restoration of serotonergic transmission but also to their ability to modulate the immune response of microglia/macrophages through specific receptors. However, it should be noted that the interpretation of these results requires considerable caution due to the pronounced sexual dimorphism in the mechanisms underlying neuropathic pain. According to current evidence, in male rodents, microglia and microglia-mediated signaling pathways (e.g., p38 MAPK) play a dominant role in the development of hypersensitivity following nerve injury. In contrast, in females, this process is largely independent of microglia and is mediated by adaptive immune cells, particularly T lymphocytes, which infiltrate the injury site and release pro-inflammatory cytokines [218,219].

Since the conclusions regarding the modulatory effects of serotonin on microglia and neuropathic pain are based on studies conducted predominantly in males, their extrapolation to females may be speculative and potentially ineffective due to the fundamentally different cellular mechanisms underlying neuropathic pain in females. Moreover, the expression and function of serotonin receptors may also differ between sexes, adding an additional layer of complexity. Thus, targeting serotonergic modulation at microglia to simultaneously reduce neuroinflammation and neuropathic pain is not a one-size-fits-all approach, but rather requires substantial adjustment to account for sex differences. Future studies should not only rigorously account for animal sex but also specifically investigate the interplay between the serotonergic system and T cells, in order to develop effective and safe therapeutic strategies for both sexes.

It has also been shown that serotonin enhances the mobility of microglia, directing them to the injury site. This effect was probably mediated by ATP: the chemotactic response of cultured microglial cells to ATP was potentiated in the presence of serotonin. The same study examined phagocytic activity: it was established that serotonin suppresses it in amoeboid and neonatal microglia, whereas in adult ramified microglia the administration of serotonin had no significant effect [220]. This may be due to the absence of stress signals, as well as to the fact that during development serotonergic signaling may serve as an indicator of the activity of synaptic contacts, which accordingly do not require elimination. Notably, it was demonstrated that blockade of signaling through the 5-HT_2B_ receptor in microglia during the early postnatal period (up to P30) reduces phagocytic activity, which leads to an increased density of dendritic spines and, consequently, to disrupted maturation of neuronal pathways. In mice with knockout of the *Htr2b* gene, disturbance of social adaptation, an abnormal response to novelty, and reduced social activity were observed [221]. Taken together, these data indicate that the effect of serotonin on microglia depends on the developmental stage and the microenvironment.

Stimulation of 5-HT_7_ receptors triggers PKA-dependent cascades that promote the expression of anti-inflammatory genes and suppress the production of pro-inflammatory mediators in macrophages/microglia [222,223]. At the level of intracellular cascades, this transition involves a switch of signaling pathways from the dominance of p38 MAPK and NF-κB to the activation of PI_3_K/Akt and cAMP/PKA signaling. The PI_3_K/Akt pathway promotes cell survival and the suppression of inflammatory activation, in part through inhibition of GSK-3β and reduction in NF-κB activity. At the same time, cAMP-dependent mechanisms enhance the transcription of anti-inflammatory genes through the CREB factor [224,225]. It has been shown that in prematurely born mice, stimulation of 5-HT_7_ receptors led to a reduction in neuroinflammation and promoted the maturation of immature oligodendrocytes [226]. This makes it possible to view stimulation of these receptors as a potentially effective strategy for enhancing endogenous remyelination after SCI and improving the recovery of sensorimotor functions.

Thus, serotonin is able to induce a phenotypic shift in microglia from a pro-inflammatory toward a reparative/immunoregulatory state; however, this process depends on a number of factors: the type of receptors activated, the stage and duration of the inflammatory response, the local concentration of serotonin, and the activity of the coupled intracellular signaling cascades. The serotonergic system acts as a regulatory hub that integrates neuronal and immune signals, but in SCI the availability of serotonin drops sharply because of the loss of descending fibers and the disruption of SERT function, which leads to compensatory supersensitivity of the tissue [227,228]. This creates special conditions under which modulation of serotonergic transmission can exert non-linear effects. A priority is to confirm that, under inflammatory conditions in SCI, targeted modulation of the serotonergic system (with 5-HT receptor agonists/antagonists or SERT inhibitors) will affect the phenotypic profile of microglia and their phagocytic activity toward myelin debris and cellular debris (which exert an inhibitory effect on the regeneration of nervous tissue), as well as functional outcomes (Figure 2).

## 5. Serotonergic Treatment Approaches After SCI

### 5.1. Pharmacological Approaches

Treatment of SCI using serotonin receptor agonists and antagonists is regarded as a promising direction, capable not only of modulating neuronal activity but also of potentially influencing the processes of remyelination and astrogliosis and reducing inflammation. The serotonergic system plays a key role in regulating the spinal neural networks that support motor, sensory, and autonomic functions, and therefore its pharmacological modulation may promote recovery after injury.

It has been shown that 5-HT_1A_ receptor agonists improve functional outcomes after SCI in rodents and humans by enhancing the activation of preserved motoneurons and promoting partial recovery of motor activity [229]. Chronic use of the 5-HT_1A_ agonist NLX-112 is accompanied by improved motor function and control of the lower urinary tract in experimental models, which is especially important in the chronic stage of SCI [133]. These effects are probably related not only to increased neuronal excitability but also to deeper plastic rearrangements in the spinal networks. In this connection, the high capacity of serotonergic neurons for regeneration and sprouting after injury makes the 5-HT system an important target for stimulating neuroplasticity [5]. Other 5-HT_1_ receptor subtypes are also of interest: for example, activation of 5-HT_1F_ receptors (the agonist LY344864) stimulates mitochondrial biogenesis and improves energy metabolism, the integrity of the blood–brain barrier, and motor function in mice after SCI [230]. Such effects may indirectly create more favorable conditions for oligodendrocyte survival and myelin restoration (Table 2).

Among 5-HT_2_ receptor agonists, quipazine attracts the most attention; in experiments on chronic spinal rats, it effectively stimulates plantar stepping, acting both on the CPG interneurons and directly on motoneurons, facilitating the output stage of the locomotor system [151]. It is important to note that 5-HT_2A_ receptor agonists such as TCB-2 demonstrate analgesic properties: in a spinal cord hemisection model, TCB-2 reduced mechanical allodynia and thermal hyperalgesia by restoring the function of the KCC2 co-transporter, which points to its potential in correcting the neuropathic pain that often accompanies SCI [231] (Table 2).

Activation of 5-HT_3_ receptors with the agonist SR 57227 leads to hyperalgesia and allodynia, accompanied by reactivation of microglia and astrocytes. Morphometric analysis of the spinal cord 2 h after intrathecal administration of SR 57227 revealed hypertrophy of glial cells in the dorsal horn (thickening of processes, enlargement of perikarya, increased staining density) as well as a significant increase in the immunoreactivity of the markers GFAP and Iba1 compared with controls [232]. Consequently, stimulation of the 5-HT_3_ receptor may not only hinder rehabilitation after SCI but also further intensify the pro-inflammatory background driven by reactive gliosis (Table 2).

The 5-HT_4_ receptor agonist tegaserod is of considerable interest. In a mouse model of compression spinal cord injury, tegaserod, initially approved for clinical use in irritable bowel syndrome and constipation, significantly improved hindlimb motor function 6 weeks after injury, increased neuronal survival, promoted sprouting into the lesion area, reduced the area and intensity of glial fibrillary acidic protein (GFAP) immunoreactivity, and stimulated axonal sprouting of catecholaminergic and serotonergic fibers. These data indicate that the neuroprotective effects of tegaserod may be related not only to activation of 5-HT_4_ receptors but also to its ability to mimic the action of polysialic acid, promoting neuritogenesis and attenuating astrogliosis [233] (Table 2).

5-HT_7_ receptor agonists, along with 5-HT_1A_ agonists, play a key role in activating the spinal locomotor network. In studies on chronic spinal rats, the 5-HT_1A_/_7_ agonist 8-OH-DPAT significantly improved interlimb coordination, facilitating CPG activity without a direct effect on motoneurons. The combined use of 8-OH-DPAT and quipazine made it possible to achieve well-coordinated weight-supported locomotion with a reduced need for exteroceptive stimulation, which underscores the potential of a multireceptor strategy [151]. Activation of 5-HT_7_ receptors with selective ligands (serdolin, MOA51) leads to a significant reduction in GFAP expression and suppression of the reactivity of microglia and astrocytes, which points to their anti-inflammatory potential. In experimental models of both inflammatory and neuropathic pain, these compounds effectively eliminate mechanical allodynia and hyperalgesia. Importantly, serdolin and MOA51, unlike pregabalin, do not induce tolerance, which makes them promising candidates for chronic therapy. Moreover, in addition to suppressing glial activation, these ligands reduce neuronal hyperactivity in the spinal cord, which provides a dual mechanism of analgesic action [234] (Table 2).

**Table 2:** Serotonin receptor agonists and their effects in SCI.

Receptor Subtype	Compound	Species	Days post SCI/Route of Administration	Effects
5-HT_1A_	NLX-112	Rat	Twice daily for 6 weeks, starting at week 2 post-contusion (T8)	Improved motor function, lower urinary tract control function (both detrusor and sphincter activities), reduced bladder weight and improved bladder morphology, reduced SCI-upregulated spinal 5-HTR_1A_ expression below the injured site [235]
	8-OH-DPAT (also agonist 5-HT_7_)	Rat	Single intravenous injection at 2 months post-complete spinal cord transection (T9–T10)	Improved hindlimb left–right alternation during bipedal walking [151]
5-HT_1F_	LY344864	Mouse	Intraperitoneal daily for 21 days, starting 1 h post-contusion (T10–12)	Improved motor function; protection of the blood–brain barrier; stimulation of mitochondrial biogenesis and enhancement of energy metabolism [230]
5-HT_2A_	TCB-2	Rat	Intraperitoneal, day 21 post-left T8 hemisection	Reduction in mechanical allodynia and thermal hyperalgesia [231]
5-HT_2_	Quipazine (with the highest affinity)	Rat	Single intraperitoneal injection on day 21 after complete transection (T9–10)	Stimulation of plantar stepping in chronic spinal rats [151]
5-HT_3_	SR 57227	Rat	Intrathecal injection following spinal nerve ligation (L5)	Hyperalgesia and allodynia; exacerbation of reactive gliosis [232]
5-HT_4_	Tegaserod	Mouse	Subdural injection 14 days, starting immediately after post-contusion	Improved hindlimb motor function, increased neuronal survival, promoted sprouting into the lesion area, reduced the area and intensity of GFAP-immunoreactivity, and stimulated axonal sprouting of catecholaminergic and serotonergic fibers [233]
5-HT_7_	Serdolin, MOA51	Mouse/Rat	Oral injection once daily for 10 days in a spinal nerve injury	Improved locomotor coordination (CPG); reduced GFAP expression and suppressed microglial and astrocytic reactivity; alleviation of mechanical allodynia and hyperalgesia [234]

Within the framework of pharmacological neuromodulation, not only activation but also blockade of individual receptor subtypes is of interest. By reducing excessive excitability and preventing the development of spasticity, antagonists can create a more favorable environment for the recovery of axons and myelin. Thus, studies in healthy people have shown that 5-HT_2_ receptor antagonists reduce the firing rate of motor units and the level of persistent inward currents during voluntary muscle contractions, which indicates their potential role in preventing spasticity and hyperexcitability [236].

Among 5-HT_2_ receptor antagonists, cyproheptadine is the most studied. In a rat model of contusion spinal cord injury, chronic administration of cyproheptadine significantly reduced the frequency of spastic behavioral reactions and decreased spinal hyperreflexia, although it had no significant effect on locomotor function. Notably, treadmill exercise combined with cyproheptadine produced no synergistic or additive effect, which points to the need for further study of optimal combined-therapy regimens [237] (Table 3).

Blockade of 5-HT_3_ receptors is regarded as a promising strategy for relieving the neuropathic pain associated with SCI. The 5-HT_3_ receptor antagonist tropisetron demonstrated a pronounced antinociceptive effect in a rat model of compression injury, which points to its potential in central neuropathic pain, especially in patients with hyperalgesia and tactile allodynia [238]. Prolonged blockade of 5-HT_3_ receptors (for example, with Y25130) reduced mechanical allodynia after SCI, which confirms the role of these receptors in pain modulation [232]. In addition, blockade of the 5-HT3 receptor was also shown to correlate with a decrease in GFAP expression [228], which can be regarded as a means of suppressing reactive astrogliosis (Table 3).

Antagonists and inverse agonists of 5-HT_6_ receptors represent a new direction in the treatment of pain syndromes in SCI. It has been shown that constitutively active spinal 5-HT_6_ receptors activate the mTOR signaling pathway, which contributes to the development of neuropathic pain and the accompanying cognitive impairments. Administration of inverse agonists of 5-HT_6_ receptors (for example, 2-(4-Fluorophenyl)-1-[(3-chlorophenyl)sulfonyl]-N-(piperidin-4-yl)-1H-pyrrole-3-carboxamide hydrochloride) prevented tactile allodynia in a rat spinal nerve ligation model by inhibiting the 5-HT_6_R-mediated Cdk5 and mTOR signaling pathways [239]. These data open new possibilities for the pharmacological correction of both pain and cognitive impairments in patients with SCI (Table 3).

It is important to emphasize that 5-HT_1A_ and 5-HT_3_ receptor antagonists can modulate the hyperexcitability of the dorsal horns of the spinal cord. In a rat model of chronic hemisection, blockade of both 5-HT_1_A and 5-HT_3_ receptors led to attenuation of the inhibitory action of exogenous serotonin, restoring the elevated evoked activity of dorsal horn neurons by 70% [240]. This indicates that combined blockade of several receptor subtypes may be an effective strategy for normalizing sensory processing after injury.

Despite their promise, the clinical translation of these approaches remains a challenging task. SCI is a multifactorial condition in which the processes of neurodegeneration, inflammation, demyelination, and spontaneous plasticity are closely intertwined. Effective therapy will probably require combined approaches that take into account the stage of injury, its severity, and the individual features of the patient. Promising options are strategies that combine pharmacological modulation with physical rehabilitation [237], electrical stimulation of the spinal cord, and cell transplantation [229]. In theory, a combination of agonists and antagonists of different 5-HT receptor subtypes may allow finer tuning of both neuronal activity and the processes of glial regeneration. Further research should be aimed at clarifying the role of individual serotonin receptor subtypes in regulating the function of oligodendrocytes, astrocytes, and microglia and at identifying optimal regimens for their pharmacological modulation.

### 5.2. Transplantation of Serotonergic Neurons or Serotonin-Producing Cells

Cell technologies open up fundamentally new possibilities in the treatment of SCI. At the core of these approaches lies the transplantation of stem cells, whose unique regenerative properties make them a promising tool for SCI therapy [241]. Owing to their multipotency, stem cells are able to differentiate into various cell types, including neurons and oligodendrocytes, and thereby replace lost cell populations [242]. The therapeutic effect is realized through a set of complementary mechanisms: neuroprotection, immunomodulation, stimulation of axonal growth, formation of new neural connections, and remyelination [243,244,245]. In addition, stem cells actively modulate the microenvironment in the lesion area: they suppress chronic inflammation, inhibit apoptotic cell death, and attenuate glial scarring, thereby creating a favorable environment for functional recovery [245,246,247].

However, despite their considerable potential, the wide introduction of cell transplants into clinical practice for the treatment of SCI is constrained by a number of fundamental and applied limitations. In the case of embryonic stem cells, unresolved ethical issues associated with the destruction of embryos come to the fore [248]. The use of induced pluripotent stem cells, obtained from the somatic tissues of the adult organism, makes it possible to circumvent this problem, but it carries a risk of teratoma and other tumor formation caused by their high proliferative activity and genetic instability [249]. An additional obstacle is the probability of immune rejection [250]. Also of critical importance is the absence of unified standardized protocols for the isolation, cultivation, and differentiation of stem cells, without which it is impossible to guarantee the reproducibility and safety of the transplanted cells. Particular attention is warranted by the fact that the microenvironment of the injured spinal cord directs the differentiation of most transplanted stem cells into glial elements rather than neurons, which substantially limits the neuroregenerative effect and makes it necessary to develop targeted protocols of neuronal differentiation [251]. Finally, for the successful translation of cell technologies into the clinic, a decisive role is played by the creation of suitable biomaterials and three-dimensional scaffolds that support the viability, spatially oriented integration, and functional maturation of the transplanted cells [252,253,254].

The main studies have focused on the transplantation of several promising cell populations: Schwann cells, neural stem cells (NSCs), oligodendrocyte precursors, olfactory cells, and mesenchymal stem cells (MSCs) [255,256,257], as well as embryonic stem cells (ESCs) and induced pluripotent stem cells (iPSCs) [258,259] (Table 4).

The main approaches to cell therapy of SCI include the transplantation of stem and glial cells. A more difficult task remains the transplantation of committed neuroblasts, in particular serotonergic neurons, since it requires the development of specialized protocols for preparing and maintaining the microenvironment needed to preserve their differentiated phenotype and their successful integration into the recipient’s injured tissue [260]. At the same time, transplantation of 5-HT neurons is potentially able to restore serotonergic neurotransmission and thereby to modulate the activity of both nerve cells (stimulating their function) and glial cells, as well as to directly replace damaged or lost neurons.

**Table 4:** Influence of transplanted cells on recovery after SCI.

Transplanted Cells	Effect	Conditions	Model Organism	References
MSCs	Enhancing the anti-inflammatory and regenerative phenotype of macrophages/microglia Suppression of the secretion of pro-inflammatory cytokines (TNF-α, IFNγ) and enhancement of the production of anti-inflammatory mediators (IL-4, IL-10) Reduction in neuropathic pain Improvement of motor functions, protection of white matter	Into the injury area 3 days after injury	Rat	[261]
		Intravenously one week after injury	Rat	[262]
		Intrathecally immediately after injury	Rat	[263]
		Intrathecal administration on days 1, 3, and 7 after injury	Rat	[264,265]
		Intrathecally immediately after injury	Human	[266]
		Intrathecally in the chronic period	Human	[267]
		Intrathecally/intraspinally in the chronic period Intrathecally in the chronic period of SCI	Human	[268,269,270]
Schwann cells and olfactory epithelium cells	Reduction of neuropathic pain Suppression of the inflammatory polarization of microglia/macrophages Restoration of bladder function Reduction in cyst size	Intraspinally 6 months after injury (around the lesion)	Human	[264,271,272]
		Intralesionally one week after injury	Rat	[273,274,275,276,277,278]
Oligodendrocyte precursor cells	Enhancement of remyelination and functional recovery	Intraspinally one week after injury	Rat	[255,279,280,281]

The researchers isolated a cell suspension from the rhombencephalic raphe nucleus of embryos and transplanted it into the completely transected spinal cord of rats. It was found that the transplants successfully engrafted, differentiated, and formed synaptic contacts in the anterior horn (the motor zone) and the intermediolateral column (the zone of the autonomic nervous system), leading to morphological reinnervation and functional improvements below the level of injury. This work showed that mature nervous tissue is able to provide guidance signals for ingrowing axons [282,283]. Other works later also showed that transplantation of embryonic serotonergic raphe neurons below the site of complete spinal cord transection promotes improvement of motor function in rats [284,285].

A number of studies exist on the transplantation of the immortalized human neuron line hNT2.19, which secretes 5-HT. The hNT2.19 cell line was selected from subclones of an embryonic carcinoma culture treated with retinoic acid and mitosis inhibitors [286,287]. hNT2.19 is characterized by uniform morphology and is often chosen because of the ease of its proliferation and differentiation in vitro, as well as the absence of oncogenic properties [288]. Transplantation of hNT2.19 cells has a positive effect on functional recovery after SCI, and its efficacy is directly related to the localization of the transplant. Thus, implantation into the subarachnoid space or the dorsal horn of the spinal cord leads to attenuation of neuropathic pain [289,290]. In a model of contusion spinal cord injury, hNT2.19 cells transplanted into the subarachnoid space completely eliminated tactile allodynia and thermal hyperalgesia, and the effect did not weaken over 8 weeks. However, no improvement in motor function was detected with this transplant localization [288].

In another series of experiments, the immortalized rat neuronal cell line RN46A-B14, derived from embryonic raphe nuclei and secreting 5-HT and BDNF, was used. It was shown that implantation of these cells directly into the injury area has no antinociceptive effect, whereas administration into the subdural space or directly into the dorsal horn of the spinal cord reduces the severity of neuropathic pain. This effect is due to the release of serotonin, which acts as an inhibitory mediator and reduces neuronal hyperexcitability [291]. After transplantation of RN46A-B14 cells, the level of 5-HT in the dorsal horns of the spinal cord was restored [292], the level of 5-HTP in the cerebrospinal fluid increased, and the hyperexcitability of neuronal membranes decreased [293]. The latter is known to be associated with tactile allodynia and thermal hyperalgesia after spinal cord injury [294]. As for motor function, implantation directly into the lesion had a positive effect. In a contusion injury model, intraspinal administration of cell transplants led to improvement of motor functions [288]. Similar results were obtained with the RN46A-B14 cell line as well: its introduction into the lesion improved motor function in rats [291].

However, despite the effectiveness of such approaches, combining cell technologies with rehabilitation methods, such as enriched environments, promotes more pronounced recovery. It is also worth noting that in the study by Eaton et al. the most pronounced recovery of motor functions was observed precisely with the combination of intraspinal transplantation of differentiated serotonergic hNT2.19 cells and environmental enrichment, applied from early time points after injury through day 56. However, unlike the overall assessment of locomotion, analysis of finer parameters of motor activity (foot rotation, number of errors when crossing a grid track) showed that the combined therapy provided no statistically significant advantage over each of the strategies individually, demonstrating only an additive rather than a synergistic effect [287]. This emphasizes that achieving a meaningful clinical result in the treatment of contusion spinal cord injury requires not merely a mechanical combination of the two approaches but also optimization of their interaction, for example through modulation of the levels of neurotrophins (BDNF), serotonergic receptors, and CPG activity. Thus, the combination of cell therapy and environmental stimulation is promising but requires further preclinical study of the mechanisms underlying the recovery of specific aspects of motor and postural function.

### 5.3. Limitations: Receptor Heterogeneity, Differences in Effects Depending on Time After Injury, and Species Specificity

Despite the promising preclinical data, the translation of serotonergic modulation into SCI therapy runs up against three fundamental barriers: receptor heterogeneity, the critical dependence of the outcome on the time window, and the species specificity of the serotonergic system. Awareness of these limitations is necessary for developing rational treatment protocols.

#### 5.3.1. Heterogeneity of 5-HT Receptors: Neuronal and Glial Targets

**Figure 3:**
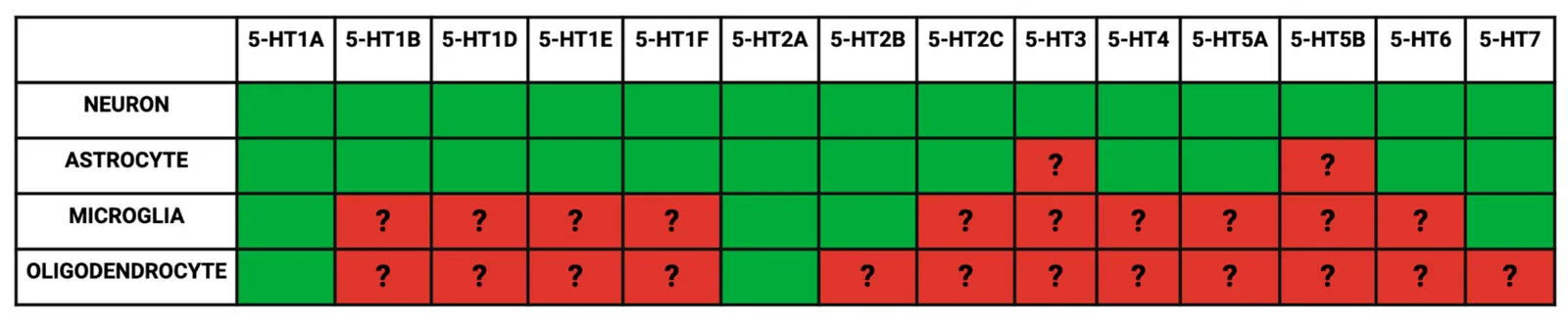
Distribution of serotonergic receptors expressed on neurons and glial cells in the CNS; a red cell with a question mark indicates that there are no data allowing a definitive statement about the absence of receptor expression.

The serotonergic system is characterized by considerable heterogeneity of the receptor apparatus expressed not only on neurons but also on glial cells (astrocytes, oligodendrocytes, microglia, and OPCs), as discussed in detail in the previous chapters. At the same time, the receptor repertoires on neurons and glia partly overlap. Thus, in the neurons of the rodent CNS the receptors 5-HT_1A_, 5-HT_1B_, 5-HT_1D_, 5-HT_1E_, 5-HT_1F,_ 5-HT_2A_, 5-HT_2B_, 5-HT_2C_, 5-HT_3_, 5-HT_4_, 5-HT_5A_, 5-HT_5B,_ 5-HT_6_, and 5-HT_7_ have been identified [115,295,296], whereas astrocytes express 5-HT_1A-F_, 5-HT_2A-2C_, 5-HT_5A_, 5-HT_6_, and 5-HT_7_, microglia express 5-HT_1A_, 5-HT_2A_, 5-HT_2B_, and 5-HT_7_, and oligodendrocytes express 5-HT_1A_ and 5-HT_2A_ (data are lacking for the other types) (Figure 3). It should be emphasized that serotonin receptor expression exhibits considerable plasticity: it is modulated in response to pathological states (including injury) and differs according to the functional specialization, cellular localization, species, age, as well as experimental conditions. Accordingly, the distribution presented in Figure 3 should be interpreted as a reference framework rather than an exhaustive representation, as it does not encompass the full range of potential dynamic alterations.

A promising task for future research is to develop a strategy for modulating the serotonergic system that takes into account the receptor profile of both neurons and glial cells. This approach is necessary because activation of the same receptors can lead to divergent effects depending on the cell type. For example, stimulation of 5-HT_2B_ receptors on microglia and astrocytes suppresses the release of pro-inflammatory cytokines and, in astrocytes, additionally promotes the secretion of BDNF. At the same time, activation of this same receptor on motoneurons can aggravate muscle spasticity, since after spinal injury the 5-HT_2B_ receptor becomes constitutively active [297]. Consequently, systemic pharmacological intervention without regard for the cellular context carries a risk of undesirable effects. In this connection, a priority remains the development of subtype-selective agonists/antagonists and the introduction of pharmacogenetic approaches that enable cell-specific modulation of the serotonergic system.

#### 5.3.2. Differences in Effects Depending on Time After Injury

The time window for modulating the serotonergic system is of critical importance in spinal injury. In the acute period after contusion injury, the concentration of serotonin in the spinal cord rises sharply (by a factor of 35–90) and normalizes within half an hour [298]. Subsequently, as the 5-HT fibers degenerate, chronic serotonergic deprivation develops, triggering compensatory plasticity of the receptors caudal to the level of injury. The dynamics of the expression of key receptors show pronounced temporal differences. An increase in the density of 5-HT_2A_ receptors on motoneurons is noted one day after injury and reaches a plateau by day 28 after complete transection of the spinal cord in rats [299]. This dynamic correlates with the development of denervation hypersensitivity and spasticity. The expression of 5-HT_2C_ receptors increases more slowly: in the gray matter of the spinal cord a significant increase is recorded by day 21, and in motoneurons by day 45. The time course of the increase in 5-HT_2C_ receptor density correlates positively with the clinical manifestations of tail spasticity in rats [300]. These data mean that a pharmacological agonist applied in the acute phase will act on a completely different receptor landscape than the same agent administered several weeks later and will accordingly produce different effects. Moreover, interventions in the first hours (for example, blockade of certain receptors) may become counterproductive in the chronic stage, when restoration of serotonergic tone is required to activate locomotor networks and modulate glial cells. Clinical protocols that do not stratify patients by SCI duration with sufficient precision may be of little effect.

#### 5.3.3. Species Specificity

Translating experimental findings from animal models to clinical practice in spinal cord injury (SCI) research remains a major challenge. Despite invaluable insights gained from rodent studies, numerous clinical trials based on promising preclinical results have failed to replicate success in humans.

A critical distinction lies in corticospinal tract (CST) anatomy and function—the primary descending motor pathway. In rodents, a well-developed dorsal CST exists alongside ventral and lateral components, whereas humans lack the dorsal CST entirely, possessing instead a predominant and significantly larger lateral/anterior CST [301]. Additionally, rat CST axons synapse primarily onto interneurons, indirectly influencing motoneurons, whereas primates and humans have direct monosynaptic corticomotoneuronal connections essential for fine motor control and dexterity [302]. This powerful direct cortical influence in humans represents an evolutionary adaptation absent in rodents, complicating translation of CST plasticity-based recovery findings.

Cellular responses are also species-specific. Chronic human lesions contain fewer reactive astrocytes compared to rodents [303,304]. While reduced astrogliosis may appear beneficial, it can create a permissive environment for aberrant axonal sprouting, potentially contributing to maladaptive plasticity associated with neuropathic pain, autonomic dysreflexia, and spasticity [305,306].

Temporal dynamics of pathophysiological processes following SCI differ considerably. Spinal shock lasts several days in rodents but weeks or longer in humans [307]. Moreover, basal metabolic rate, heart rate, and respiratory rate in rats are several times higher than in humans, with genomic and inflammatory responses proceeding 30–50 times faster in rodents [307]. Consequently, the therapeutic window for interventions is compressed in animal models, and treatment timing cannot be directly extrapolated to humans.

These anatomical and physiological differences extend to neurotransmitter systems modulating spinal motor circuits. Although rodents, primates, and humans share common features—5-HT-positive projections surrounding ventral horn motoneurons and comparable 5-HT_1A_ receptor expression [308]—the organization of serotonergic innervation differs fundamentally. In primates, serotonergic innervation is perisynaptic, with terminals forming direct contacts on motoneuron somatic membranes, enabling powerful and rapid influence. By contrast, in rodents and cats, an extrasynaptic (volume transmission) type predominates, with terminals located in the neuropil between motoneurons, modulating locomotor networks via mediator diffusion [309]. This fundamental divergence in synaptic architecture cautions against direct extrapolation of serotonergic modulation results from rodents to clinical applications and underscores the need for preclinical validation in primate models more closely resembling human neuroanatomy.

In conclusion, successful translation of SCI therapies requires comprehensive understanding of interspecies differences in anatomy, physiology, and temporal dynamics. While rodents remain essential for mechanistic studies, validating therapeutic strategies in non-human primates—which more closely mirror human CST organization and serotonergic innervation—is a crucial step before clinical trials. Furthermore, awareness of the three principal limitations discussed above—receptor heterogeneity, temporal dependence of effects, and species specificity—underscores the need for precisely targeted, carefully timed, and species-appropriate approaches to serotonergic modulation in the treatment of SCI. Only by integrating these considerations into experimental design and therapeutic development can we bridge the translational gap and improve the likelihood of clinical success.

## 6. Conclusions

Literature analysis indicates that the serotonergic system represents a multitarget platform for modulating the glial component in SCI. Through 5-HT_1A_, 5-HT_2A_, 5-HT_2B_, and 5-HT_7_ receptors, serotonin promotes oligodendrocyte maturation, restricts proinflammatory astrocyte transformation, and shifts microglia toward a reparative phenotype. Of particular interest is 5-HT_2_B-mediated activation of the PI_3_K/AKT pathway and suppression of the NLRP3 inflammasome, which provide direct pathways for inducing a neuroprotective glial phenotype.

Although some of these effects have been demonstrated in isolated cultures or in the context of other pathologies, this review establishes a theoretical framework for translating these mechanisms into SCI therapy. The limitations—receptor heterogeneity, temporal dynamics, sex and species specificity—do not deny the promise of this approach; rather, they define key parameters for therapeutic design, including selective ligands, timed administration, and validation in non-human primates with consideration of sex differences.

The main conclusion is that serotonergic modulation of glia is experimentally substantiated, and its potential substantially outweighs the risks. The priority for future research is testing 5-HT_2B_ and 5-HT_7_ agonists in SCI models, using hNT2.19 and RN46A-B14 cell lines, with assessment of motor recovery, remyelination, and myelin debris phagocytosis. This work sets a trajectory for preclinical and clinical development, positioning the serotonergic system as a realistic target for combined neurorehabilitative therapy.

## Figures and Tables

**Table 3:** Serotonin receptor antagonists and their effects in SCI.

Receptor Subtype	Compound	Species	Days Post SCI/Route of Administration	Effects
5-HT_2_	Cyproheptadine	Rat	Intraperitoneal daily for 2 weeks, starting at week 4 post-contusion	Reduction in the frequency of spastic behavioral reactions and spinal hyperreflexia; no effect on locomotion [237]
5-HT_3_	Tropisetron	Rat	Single intrathecal injection at 4 weeks after post-contusion (T6–8)	Reduction in hyperalgesia and tactile/mechanical allodynia [238]
	Y25130	Rat	Intrathecal in model spinal nerve ligation (L5)	Reduction in thermal hyperalgesia and mechanical allodynia [232]
5-HT_6_	2-(4-Fluorophenyl)-1-[(3-chlorophenyl)sulfonyl]-N-(piperidin-4-yl)-1H-pyrrole-3-carboxamide hydrochloride	Rat	Single intraperitoneal injection on day 14 after spinal nerve ligation (L5)	Reduction in tactile allodynia [239]

## Data Availability

No new data were created or analyzed in this study. Data sharing is not applicable to this article.

## Abbreviations

5-HT	5-Hydroxytryptamine (serotonin)
5-HTP	5-Hydroxytryptophan
5-HTR	5-Hydroxytryptamine receptor
8-OH DPAT	8-Hydroxy-2-(di-n-propylamino)tetralin
AADC	Aromatic L-amino acid decarboxylase
AC	Adenylate cyclase
ADAR2	Adenosine deaminase acting on RNA 2
ADP	Adenosine diphosphate
Akt	Protein kinase B
AMPA	α-Amino-3-hydroxy-5-methyl-4-isoxazolepropionic acid receptor
AQP4	Aquaporin-4
ARF	ADP-ribosylation factor
ATP	Adenosine triphosphate
BCL2	B-cell lymphoma 2
BDNF	Brain-derived neurotrophic factor
BMP	Bone morphogenetic protein
C1q	Complement component 1q
C3	Complement component 3
cAMP	Cyclic adenosine monophosphate
Caspr	Contactin-associated protein
Cav-1	Caveolin-1
CaV1.2	L-type voltage-gated calcium channel 1.2
CC-1	Adenomatous polyposis coli clone CC-1
CCL2	C-C motif chemokine ligand 2
CCR2	C-C motif chemokine receptor 2
CD68	Cluster of differentiation 68
Cdc42	Cell division control protein 42
Cdk5	Cyclin-dependent kinase 5
CNPase	2′,3′-Cyclic nucleotide 3′-phosphodiesterase
CNS	Central nervous system
cPLA2 IVA	Cytosolic phospholipase A2 group IVA
CPG	Central pattern generator
CREB	cAMP response element-binding protein
CSPGs	Chondroitin sulfate proteoglycans
CX3CR1	CX3C chemokine receptor 1
Cx43	Connexin 43
CXCL	C-X-C motif chemokine ligand
Cyb5r3	Cytochrome b5 reductase 3
DAG	Diacylglycerol
DAMPs	Damage-associated molecular patterns
DIO3 (D3)	Deiodinase 3
DNA	Deoxyribonucleic acid
DR	Death receptor
EAAT	Excitatory amino acid transporter
Echs1	Enoyl-CoA hydratase, short chain 1
ERK	Extracellular signal-regulated kinase
ESCs	Embryonic stem cells
FAK	Focal adhesion kinase
FGF2	Fibroblast growth factor 2
GalC	Galactocerebroside
GDNF	Glial cell line-derived neurotrophic factor
GFAP	Glial fibrillary acidic protein
Gfpt2	Glutamine-fructose-6-phosphate transaminase 2
GLAST	Glutamate aspartate transporter (EAAT1)
GLT-1	Glutamate transporter 1 (EAAT2)
GluK2	Glutamate ionotropic receptor kainate type subunit 2
GPCRs	G-protein-coupled receptors
GSK-3β	Glycogen synthase kinase 3 beta
Hadh/Hadha	Hydroxyacyl-CoA dehydrogenase subunits
HK2	Hexokinase 2
HMGB1	High mobility group box 1
Htr2b	5-Hydroxytryptamine receptor 2B gene
Iba1	Ionized calcium-binding adapter molecule 1
Icam1	Intercellular adhesion molecule 1
Id2/Id4	Inhibitor of DNA binding 2/4
IDO	Indoleamine 2,3-dioxygenase
IFN-γ	Interferon gamma
IGF-1	Insulin-like growth factor 1
IL	Interleukin
IP_3_	Inositol 1,4,5-trisphosphate
iPSCs	Induced pluripotent stem cells
JAK/STAT	Janus kinase/signal transducer and activator of transcription
KA	Kainate receptor
KCC2	Potassium-chloride cotransporter 2
Lgals1	Galectin-1
Lif	Leukemia inhibitory factor
LPS	Lipopolysaccharide
Ly6C	Lymphocyte antigen 6 complex, locus C
MAG	Myelin-associated glycoprotein
MAO A	Monoamine oxidase A
MAP1B-LC1	Microtubule-associated protein 1B light chain 1
MAPK	Mitogen-activated protein kinase
MBP	Myelin basic protein
MCP-1	Monocyte chemoattractant protein-1
MMP-9	Matrix metalloproteinase 9
mRNA	Messenger ribonucleic acid
MSCs	Mesenchymal stem cells
mTOR	Mechanistic target of rapamycin
NF-κB	Nuclear factor kappa-light-chain-enhancer of activated B cells
NG2	Neural/glial antigen 2
NLRP3	NOD-, LRR- and pyrin domain-containing protein 3
NMDA	N-methyl-D-aspartate receptor
NO	Nitric oxide
Nogo	Neurite outgrowth inhibitor
NOX	NADPH oxidase
NSCs	Neural stem cells
NT-3	Neurotrophin-3
Olig1/Olig2	Oligodendrocyte transcription factor 1/2
OMgp	Oligodendrocyte myelin glycoprotein
ONOO^−^	Peroxynitrite
OPCs	Oligodendrocyte precursor cells
P2RY12	Purinergic receptor P2Y12
P2X/P2Y	Purinergic receptors
PI3K	Phosphoinositide 3-kinase
PIP2	Phosphatidylinositol 4,5-bisphosphate
PKA	Protein kinase A
PKC	Protein kinase C
PLC	Phospholipase C
PLD	Phospholipase D
PLP	Proteolipid protein
PTPσ	Protein tyrosine phosphatase sigma
QUIN	Quinolinic acid
RIP	Receptor-interacting protein (mature oligodendrocyte marker)
RNS	Reactive nitrogen species
ROS	Reactive oxygen species
SCI	Spinal cord injury
SERT	Serotonin transporter
SSRI	Selective serotonin reuptake inhibitor
TCAs	Tricyclic antidepressants
TCB-2	(4-Bromo-3,6-dimethoxybenzocyclobuten-1-yl)methylamine
TGF-β	Transforming growth factor beta
TLR	Toll-like receptor
TNF-α	Tumor necrosis factor alpha
TPH2	Tryptophan hydroxylase 2
TREM2	Triggering receptor expressed on myeloid cells 2
TRPC1	Transient receptor potential canonical 1
Vcan	Versican
VEGF	Vascular endothelial growth factor
Vgf	VGF nerve growth factor inducible
Vim	Vimentin
